# Could a rabies incursion spread in the northern Australian dingo population? Development of a spatial stochastic simulation model

**DOI:** 10.1371/journal.pntd.0009124

**Published:** 2021-02-12

**Authors:** Vanessa Gabriele-Rivet, Michael P. Ward, Julie Arsenault, David London, Victoria J. Brookes

**Affiliations:** 1 Sydney School of Veterinary Science, Faculty of Science, The University of Sydney, Camden, New South Wales, Australia; 2 Département de pathologie et microbiologie, Faculté de médecine vétérinaire, Université de Montréal, Saint-Hyacinthe, Québec, Canada; 3 Physique des Particules, Faculté des arts et des sciences, Université de Montréal, Montréal, Québec, Canada; 4 School of Animal and Veterinary Sciences, Faculty of Science, Charles Sturt University, Wagga Wagga, New South Wales, Australia; 5 Graham Centre for Agricultural Innovation (NSW Department of Primary Industries and Charles Sturt University), Wagga Wagga, NSW, Australia; US Department of Agriculture, UNITED STATES

## Abstract

Australia, home to the iconic dingo, is currently free from canine rabies. However northern Australia, including Indigenous communities with large free-roaming domestic dog populations, is at increased risk of rabies incursion from nearby Indonesia. We developed a novel agent-based stochastic spatial rabies spread model to evaluate the potential spread of rabies within the dingo population of the Northern Peninsula Area (NPA) region of northern Australia. The model incorporated spatio-temporal features specific to this host-environment system, including landscape heterogeneity, demographic fluctuations, dispersal movements and dingo ecological parameters—such as home range size and density—derived from NPA field studies. Rabies spread between dingo packs in nearly 60% of simulations. In such situations rabies would affect a median of 22 dingoes (approximately 14% of the population; 2.5–97.5 percentiles: 2–101 dingoes) within the study area which covered 1,131 km^2^, and spread 0.52 km/week for 191 days. Larger outbreaks occurred in scenarios in which an incursion was introduced during the dry season (vs. wet season), and close to communities (vs. areas with high risk of interaction between dingoes and hunting community dogs). Sensitivity analyses revealed that home range size and duration of infectious clinical period contributed most to the variance of outputs. Although conditions in the NPA would most likely not support a sustained propagation of the disease in the dingo population, due to the predicted number of infected dingoes following a rabies incursion and the proximity of Indigenous communities to dingo habitat, we conclude that the risk for human transmission could be substantial.

## Author summary

Although Australia is free from canine rabies, an incursion from nearby rabies-infected Indonesian islands is a realistic threat. The ubiquitous presence of dingoes in the wild, in association with large populations of free-roaming domestic dogs from northern Australian Indigenous communities, increases the risk of a rabies outbreak. Using a newly developed simulation model, we investigated the potential spread of rabies in a northern Australian dingo population. Nearly 60% of model simulations resulted in more than one pack infected. When spread did occur, outbreaks affected a median of 22 dingoes (an estimated 14% of the population in this area). The duration of infection, proportion of the population infected and spatial spread of the outbreak was greatest when rabies was introduced during the dry season and close to communities. Our results demonstrate that an incursion of rabies into the northern Australian dingo population would likely lead to a rabies outbreak, which would in turn pose a substantial threat to Indigenous communities in northern Australia.

## Introduction

Canine rabies is a vaccine-preventable viral disease, estimated to cause 59 000 human deaths annually, mostly occurring in Asia and Africa [1]. Although domestic dogs account for most canine rabies transmissions to humans [2], rabies can be maintained within a wildlife-mediated cycle involving wild canidae populations [3–6]. A feature of rabies is the behavioral change observed in clinically affected hosts, which can be classified as dumb (paralytic) and furious (hyperactive) forms. Behavior associated in animals affected with the dumb form includes restricted movement (for example, remaining confined to a den) whereas animals affected by the furious form might display signs of increased agitation leading them to roam considerable distances, as observed in rabies-infected foxes [7]. These rabies-induced behaviors most likely have an impact on contact patterns between individuals and hence disease dynamics [8].

Although Australia is historically free from canine rabies, northern Australia faces an increased risk of canine rabies introduction due to the recent eastern spread of the disease through the Indonesian archipelago, with outbreaks occurring in previously rabies-free Indonesian islands such as Flores, Ambon, Sumbawa and Bali [9–12]. The illegal boat transportation of a latent rabies infected domestic dog from Indonesia into a community in northern Australia is considered to be the most likely route of entry of rabies [13], similar to the introduction of rabies in Bali [14]. The northern Australian coast is particularly vulnerable to the establishment of rabies: this sparsely populated area contains socio-economically disadvantaged remote Indigenous communities with cultural practices that include free-roaming domestic dogs [15]; in addition, there is limited access to healthcare and veterinary care services [16].

The Australian wild dog population, composed of dingoes, feral domestic dogs and dingo-dog hybrids (collectively referred to as ‘dingo’ hereafter), is widespread across mainland Australia, inhabiting a large variety of habitat types and climate zones, including equatorial northern Australia [17]. Considering a rabies incursion in the Indigenous communities of northern Australia, the subsequent spillover of the disease from domestic dogs to dingoes is a plausible scenario, because there is evidence of opportunities for interactions between both populations [18]. For instance, in a camera-trap study conducted in the Northern Peninsula Area (NPA) of Queensland, a substantial temporal overlap of daily activity patterns was found between dingoes and free-roaming domestic dogs [19]. Furthermore, hunting expeditions with domestic dogs from northern Australian Indigenous communities create contact opportunities between hunting dogs and dingoes, increasing the risk of disease transmission at the wild-domestic interface in areas far away from the communities [20].

Infectious disease spread models play an important role in providing insight to inform decision-making and therefore efficiently control disease spread [21]. Our understanding of rabies transmission dynamics in wild animal populations, including foxes (*Vulpes vulpes*) [22] and raccoons (*Procyon lotor*) [23], has been improved by disease spread models. In Australia, the spread of rabies has been modelled in domestic dog populations from different environmental settings, including Indigenous communities in equatorial northern Australia [8,24,25]. Rabies spread was simulated within and between populations of dingoes and free-roaming domestic dogs of the mid-eastern coast of Australia using a simple stochastic state-transition model [26]. Only one model (a stochastic spatial transmission network model) has been developed to evaluate the spread of canine rabies within the dingo population in northern Australia [27]. In this model, dingo density was an influential parameter in determining whether a canine rabies epidemic would occur. However, model parameters were based on limited population data which was not specific to this region. Since this model, a recent camera-trap study conducted in equatorial northern Australia has generated spatio-temporal ecological data for the northern Australian dingo population [19], including dingo movement and density estimates, which are fundamental drivers for wildlife disease spread [21,28].

The objective of this study was to evaluate potential rabies spread within dingoes in the NPA—using parameters relevant to the northern Australian dingo context—as the first step in assessing the potential impact of a rabies incursion into the dingo population on the risk to human populations. We aimed to estimate the probability of rabies spreading between packs and, in such an event, the magnitude (in terms of duration, proportion of packs infected and number of dingoes infected) and spatial spread of rabies, considering the geographical and seasonal characteristics of the area and the ecology of the dingo population. In addition, we explored the impact of the location and season of incursion on the predicted outbreak and used global sensitivity analysis to evaluate which input parameters contribute most to variance of the model outcomes.

## Methods

### Study area

**Fig 1:**
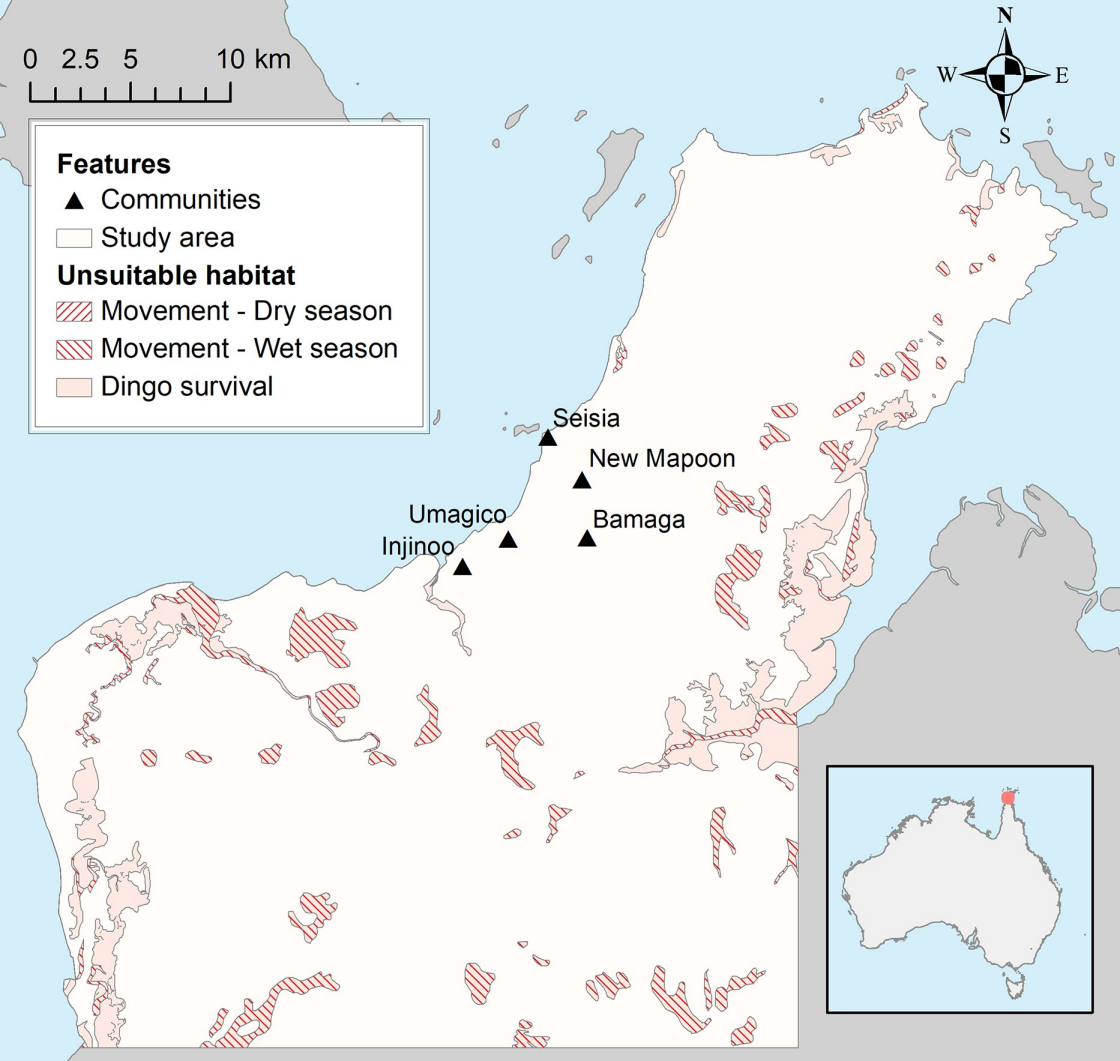
Location of the study area in the Northern Peninsula Area (NPA) of Queensland, Australia. Location of the study area and unsuitable habitat types for dingoes’ long-term survival and for movement of dingoes during the dry and wet seasons used in a spatial rabies spread model within the dingo population in the NPA.

Rabies spread was modelled in the dingo population in the Northern Peninsula Area (NPA: 10° 53′ 16″ S, 142° 23′ 16″ E) of Queensland, Cape York, on the northern coast of Australia (Fig 1). Five remote Indigenous communities are located within the NPA (Bamaga, Seisia, New Mapoon, Injinoo and Umagico), ranging from 260 [Seisia] to 1164 [Bamaga] residents [29], and contain a relatively large population of free-roaming domestic dogs (542 estimated roaming dogs [15]). Two contrasting seasons occur in this equatorial climate zone [30]; a dry season (May to October) characterised by little rainfall throughout the area (mean monthly precipitation varying between 7mm and 60mm for the period 1960–2020), and a wet season (November to April) with high levels of humidity and heavy rainfall (mean monthly precipitation varying between 50mm and 430mm for the period 1960–2020; [31]). Vegetation types include rainforest, open heath, eucalypt woodland, mangroves and wetlands [32]. Based on the Map Grid of Australia 1984 Transverse Mercator projection system, zone 54, the study area was bound to the south by the northing coordinate 8,770,000 m and on the east side by easting coordinate 662,000 m to ensure it matched the dingo population sampled, and the corresponding ecological parameters that were obtained in our previous field study [19].

### Model structure

An agent-based spatial model was implemented in Python version 3.7 [33] to simulate rabies transmission within the dingo population on a daily time step. The maximum duration for each simulation was 3 years. A simulation was defined as the execution of the model, including the creation of a new dingo population and, subsequently, rabies transmission within the population. The dingo population size in a simulation fluctuated over time throughout a given year, according to natural death and birth in the population. The centroid points of all dingo packs were randomly distributed across the study area. All members of a pack shared a common home range center; however, each dingo’s home range distribution varied independently from other dingoes in the same pack. This home range distribution represented the probability of finding a dingo around its centroid point, which was described by a bivariate Normal distribution. Dingoes could relocate to a newly vacant home range area at each time-step, according to a daily probability of relocation. Seasonal variations in terms of density and home range size were integrated into the model, based on results previously found in our field study [19]. Since young puppies are confined to the den for the first few months of life, their role in disease transmission would most likely remain negligible and, therefore, dingoes younger than 4–6 months of age were not included in the dingo population of the model.

Disease transmission was initiated by selecting one dingo to be the first latently infected individual (primary case), which was assumed to have contracted rabies from a domestic dog. The initiation of infection could occur at different times in the year and at different locations throughout the study area. Rabies infection in individual dingoes followed five stages: Susceptible (S)–Latent (E)–Infectious Pre-clinical (I_1_)–Infectious Clinical (I_2_)–Dead (R). The infection was assumed to be 100% fatal. When a dingo was infectious (I_1_ or I_2_), the daily probability of contact between this dingo and all other susceptible dingoes in the population was calculated at each time-step based on the spatial overlap of home range distributions. Transmission of rabies was dependent on the probability of bite by the infectious dingo given contact between the pair of dingoes and the probability of successful rabies transmission to the susceptible dingo, given a bite. A dingo in the infectious clinical stage (I_2_) exhibited either the furious or dumb form, which increased or decreased the probability of bite and the standard deviation of the home range distribution, respectively.

#### Initial population size

The initial population size on the first day of each simulation varied according to the time at which initiation of infection occurred, that is either the beginning of the dry season, the beginning of the second half of the dry season, the beginning of the wet season and the beginning of the second half of the wet season (details of population calculations can be found in S1 Appendix). At the start of each simulation, dingoes were allocated into packs of varying sizes (see section *Parameterisation*), until the initial calculated population size was achieved. The number of packs for a given simulation was therefore determined by the initial population size and the random allocation into packs.

#### Seasonal population fluctuations due to natural death and birth

Each dingo had an equal daily probability of dying due to natural causes, *PD*_*daily*_ described by

$${PD}_{daily}=1-e^{-\delta }$$ (1)

in which *δ* corresponds to the death rate and remains constant throughout the duration of a given simulation, regardless of season.

Consistent with previous studies [34,35] and field data in the NPA [19], the introduction of new independent dingoes into the population (i.e. when newly born dingoes of 4–6 months of age become more active and roam away from their den) occurred annually during the first half of the wet season (starting in November). Dingoes were introduced into the model throughout this 90-day period according to a daily probability of introduction equal to a constant, with a zero probability for all other days of the year.

The value of *δ* which satisfied the condition of population stability from one year to another was computed numerically by solving a non-linear equation (Eq 14 in S1 Appendix) that accounted for natural deaths, the introduction of independent dingoes into the population and the variation in the mean densities from the dry and wet seasons. Yearly fluctuations in population size occurred due to the stochasticity in birth and natural death events.

#### Spatial allocation of packs and pack size fluctuations

A polygon shapefile was created in ArcGIS version 10.5 (ESRI, Redlands, CA), delimiting the boundaries of our study area (see section *Study area*) [36]. A dataset of 31,976 points (referred to as ‘entire grid points’ hereafter), each point separated by a distance of 200 meters forming a rectangular grid, was built in SAS version 9.4 (SAS Institute Inc., Cary, NC) and overlaid within the land covered by the shapefile. To define the area in which home range centroid points of packs could occur, all points located within unsuitable habitat types for dingoes’ long-term survival—including mangroves, watercourse areas [37], marine swamps, saline coastal flats, swamps, lakes, foreshore flats [38] and ocean—were removed. Additionally, to ensure that all dingoes had most of their home range areas inland, points within a distance of 1.4 km from the ocean coast (i.e. corresponding to the lowest value of our range of home range radii sampled in our model) were removed from the dataset. This resulted in a total of 24,804 points representing the potential location of home range centroids of packs (referred to as ‘potential pack points’ hereafter).

At the start of a simulation, for each pack of dingoes, one point was randomly selected among all ‘potential pack points’, located at least 1.4 km from all points that were previously assigned to packs, to represent the location of the pack’s home range centroid point. This minimum distance condition between packs reduced biologically implausible clustering across the study area, and is consistent with a study of a population of dingoes in Western Australia with density estimates similar to our target population (0.05 and 0.22 dingoes/km^2^; [39,40]) which found that spatial overlap of dingo territories is limited.

It is important to note that the location of packs remained fixed throughout a simulation and the total number of packs at any given time during a simulation could not exceed the initial number of packs. That is, the seasonal population size fluctuations within each simulation were solely related to variation in the number of dingoes within each pack location. This is consistent with Thomson et al. [39] who observed an increase in density in a dingo population due to an increase in pack size rather than pack numbers. The home range centroid point of a pack could become ‘empty’ during a simulation due to relocation of dingoes outside the pack or death of members, but could then repopulate following relocation of a single dingo into the vacant area or introduction of new independent dingoes in that area. Each new young independent dingo introduced into the model was assigned to a pack randomly, regardless of pack size (empty to large).

#### Movement network

The distance between the centroid points of each pair of packs is required to calculate the probability of a dingo relocating to a new area and the probability of two dingoes being in contact (See following sections *Probability of relocation* and *Probability of contact*), and were pre-calculated for each season for use in simulations. Using the ‘entire grid points’, a network of lines was created by connecting each point to its 32 nearest neighboring points in all directions (i.e. the largest number of connections which reflected an optimal balance between computational time and sufficient representation of the actual shortest distance between each pair of points). These connections represented the edges in which dingoes were allowed to travel from one point to another within the resulting network (See Fig E in S1 Appendix). The portion of the lines overlapping unsuitable habitats for dingoes’ movements during the dry and wet seasons were removed from the network, resulting in a Network dataset for each season. Dingoes were therefore not allowed to cross these unsuitable habitats, but rather circumvent these obstacles, resulting in a larger distance between points. Unsuitable habitat types for dingoes’ movements during the wet season included only swamps, lakes, foreshore flats, watercourse areas and ocean, assuming that dingoes may travel across all other habitat types. For the dry season, swamps were considered as a suitable habitat type for movement, as this wetland type would often dry-out during the dry season in the study area. The shortest path between each pair of the ‘potential pack points’ was found for each seasonal network dataset, by allowing the path to go through any connecting vertices or any point of line intersection, using the Network Analyst extension functions of ArcGIS. This resulted in two pre-calculated distance matrices (i.e. dry and wet seasons) between all pairs of ‘potential pack points’.

#### Home range distribution

It was assumed that the probability of finding a dingo around its centroid point was described by a circular bivariate (2 dimensional) normal distribution, specific to the home range size of each individual dingo. Hence, the home range distribution *P*_*i*_ *(x*, *y)* for dingo *i*, which is a probability density function with dimensions of 1/(distance)^2^, was described by the following equation:

$$P_{i}(x,y)=\frac{1}{2\pi \sigma_{i}^{2}}exp\left(-\frac{x^{2}+y^{2}}{2\sigma_{i}^{2}}\right)$$ (2)

When integrated over a defined space (for example, a circle of radius *R* from the centroid point), we obtain the probability of finding the dingo within that area. Integrating over the entire space results in a probability equal to one (i.e. there is 100% chance of finding the dingo somewhere). According to our field study [19], the home range sizes of dingoes in the NPA vary between half-seasons. Therefore, each dingo was assigned four randomly selected 95% home range size values (one for each half-season). We assumed that each dingo’s home range size corresponded to the area in which there is a 95% probability of finding the dingo. That is, the dingo is found 95% of the time within a circle of radius *R*_*95*_ equal to the size of its home range and ventures outside of its home range only 5% of the time. Using this assumption, the standard deviation (*σ*) can be calculated as a proportion of *R*_*95*_ (*0*.4*R_*95*_; details of calculations found in S1 Appendix). This standard deviation parameter describes the size of the home range distribution of each dingo at each half-season, and therefore the extent of movement around its home range centroid: a larger *σ* represents a home-range in which a dingo may roam further away while a smaller *σ* reflects a dingo that is confined to movements near the den.

#### Probability of relocation

Dispersal has been described as the action of a dingo relocating permanently beyond the boundaries of its usually occupied area and occurs more readily when areas become vacant [40,41]. Within the model, home range centroids could become vacant following natural death, death due to rabies, or relocation of all members of the pack and could be recolonized by the dispersal of one single individual from another pack into the vacant area. Relocation of a dingo to a vacant area occurred when the following criteria were met: 1) a vacant home range centroid was located beyond the 95% home range area of the dingo, in accordance with the definition of dispersal and 2) the dingo found itself within an area of approximately 4 km^2^ around the location of the empty home range centroid. The probability of dingo *i* (with its standard deviation *σ*_*i*_) relocating at a new centroid point of distance *d* is therefore equal to the probability that the dingo finds itself in the vicinity of the vacated point, if this point is located beyond the 95% home range area of the dingo. By integrating the home range distribution over this approximate 4 km^2^ area, the probability of relocation can be described by (details of calculations can be found in S1 Appendix):

$${PR}_{i\_daily}=1-\sqrt[{n_{i}}]{1-\frac{1}{\pi }Arctan(\frac{1}{d})(exp(-\frac{{\left(d-1\right)}^{2}}{2\sigma_{i}^{2}})-exp(-\frac{{\left(d+1\right)}^{2}}{2\sigma_{i}^{2}}))}$$ (3)

With *d > R*_*95*_

The parameter *n*_*i*_ corresponds to the number of days needed for the dingo to explore most of its home range area, and therefore the time needed to build its home range distribution (referred to as the “number of home range days” hereafter, see section *Number of days to cover the home range area* below). For a given home range size, a larger number of home range days implies that the dingo will cover a smaller proportion of its home range area per day, hence diminishing its level of roaming activity. The daily probability of relocation is therefore also expected to decrease with the number of home range days, in accordance with Eq 3.

#### Probability of contact

With the assumption that dingoes use space independently of each other, the probability of contact between two dingoes from different packs is found by computing the degree of spatial overlap of their home range distributions. Consequently, we defined the probability of contact between dingo *i* and dingo *j* (each with its own standard deviation, *σ*, and number of home range days, *n*), both centroid points separated by a distance *d*, as follows (details of calculations found in S1 Appendix):

$$PC_{ij\_daily}=1-\sqrt[{n_{ij}}]{1-\frac{2\sigma_{i}\sigma_{j}}{\sigma_{i}^{2}+\sigma_{j}^{2}}\exp\left(-\frac{d^{2}}{4(\sigma_{i}^{2}+\sigma_{j}^{2})}\right)}$$ (4)

in which $n_{ij}=\frac{n_{i}+n_{j}}{2}$, the average number of home range days.

**Fig 2:**
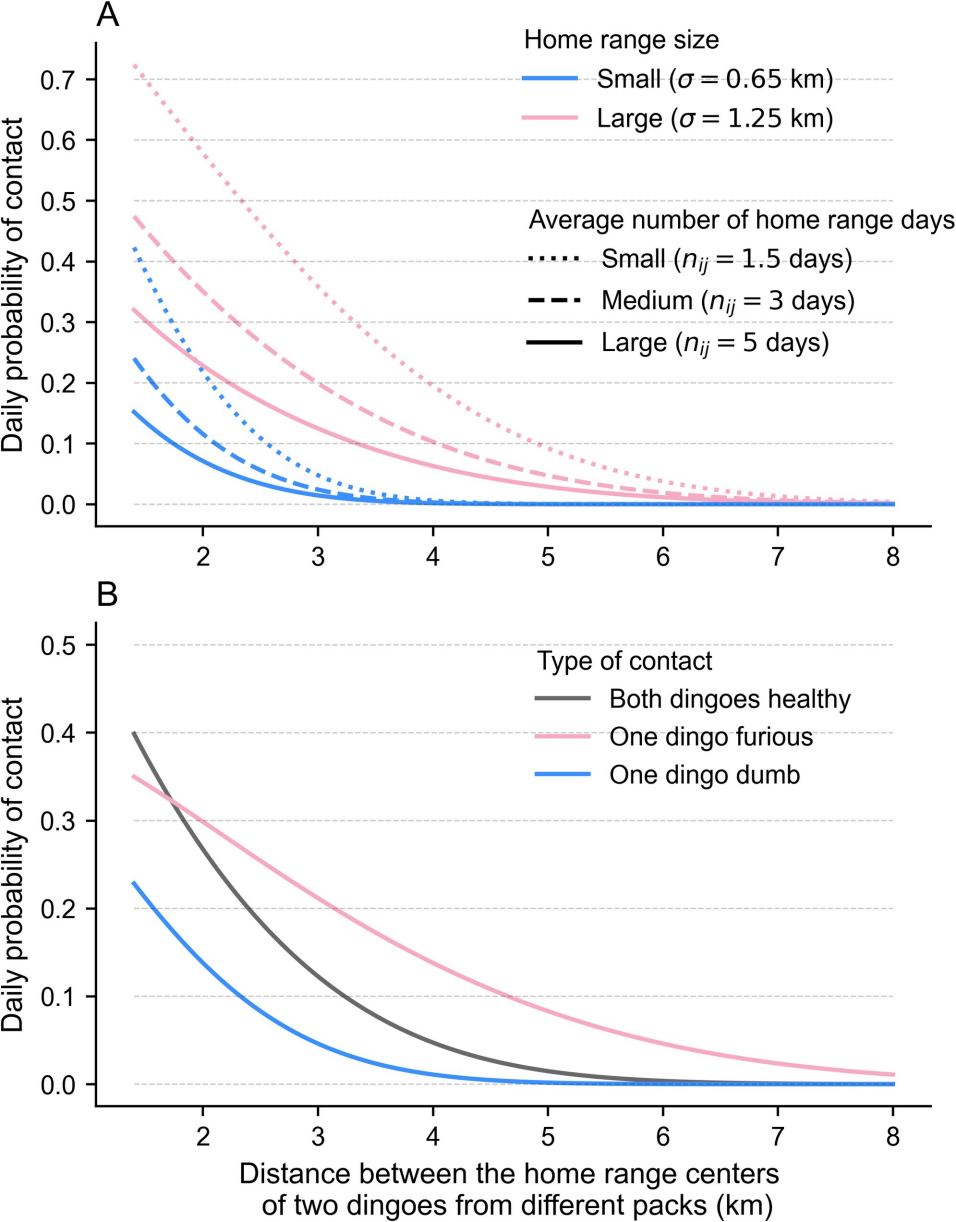
Proposed daily probability of contact of two dingoes from different packs according to distance. A) Influence of the home range size and number of home range days on the daily probability of contact between a pair of non-clinical dingoes of different packs. For illustrative purposes, the two dingoes in each pair (i.e. each curve) occupy home range areas of equal size, either small (most likely value from the second half of the wet season) or large (most likely value from the second half of the dry season). B) Influence of rabies-induced home range size changes on the probability of contact between a pair of dingoes of different packs. In this example, the healthy dingoes (grey curve) both have a standard deviation, *σ*, equal to 1.0 km, with an average number of home range days, *n*_*ij*_, equal to 3. The pink and blue curves represent the revised probability of contact if one of the individuals becomes clinically infectious with the furious and dumb forms, respectively, applying the maximum and minimum inflation factors on standard deviations (i.e. factor of 2 and 0.5, respectively), with the number of home range days unchanged.

According to this formula, the probability of contact decreased as the distance between the centroid points increased (*PC*_*ij_daily*_ *→ 0* as *d → ∞*). Fig 2A illustrates the daily probability of contact for dingoes of different packs as a function of distance between packs, in relation to variations in *σ* and *n*_*ij*_ values. When comparing pairs of dingoes with a similar *n*_*ij*_ value, the probability of contact is always higher for the pair of dingoes with the largest home range sizes, as they exhibit a higher level of roaming activity over a larger area. Pairs of dingoes with the highest level of roaming activity (Large home range sizes along with a small *n*_*ij*_ value) will have the highest probability of contact. It was assumed that two dingoes from the same pack have a 100% chance of daily contact (*PC*_*ij_daily*_ *= 1*), regardless of their respective standard deviations.

### Parameterisation

#### Density and home range

The distributions of the parameter estimates used in the model for density and home range size were based on our recent camera-trap study of the dingo population in the NPA [19]. The mean estimate of the density for the dry season as well as the home range sizes for all four half-seasons were defined as the modes of PERT distributions, with the lower and higher confidence interval values assigned as minima and maxima (Table 1). At the start of each simulation, the mean density during the wet season was obtained by multiplying the selected mean density during the dry season by a factor of 1.089, consistent with the results of our field study.

**Table 1:** Parameter values used in a spatial model to simulate rabies spread in the dingo population in the Northern Peninsula Area of Queensland, Australia.

Parameters	Distribution	Values	GSA range	References
**Dingo ecology parameters**				
Mean density (dingoes/km^2^)				
Dry season	PERT (min, ML, max)	0.127, 0.135, 0.144	0.127–0.144	[19]
Wet season		1.08 X mean density of dry season		
Home range (km^2^)			6.4–33.8	[19]
Dry season–first half	PERT (min, ML, max)	22.6, 25.3, 28.4		
Dry season–second half	PERT (min, ML, max)	26.0, 29.4, 33.8		
Wet season–first half	PERT (min, ML, max)	18.5, 21.5, 24.9		
Wet season–second half	PERT (min, ML, max)	6.4, 8.0, 9.5		
Pack size (dingoes)			1–9	[19,40,42]
Dry season–first half	PERT (min, ML, max)	1, 4.1, 7		
Dry season–second half	PERT (min, ML, max)	1, 3.6, 7		
Wet season–first half	PERT (min, ML, max)	1, 3.2, 7		
Wet season–second half	PERT (min, ML, max)	1, 4.6, 7		
Number of days needed for a dingo to explore its home range area	Uniform (min, max)	1.5–5	1.5–5	Author’s assumption, [43,44]
Home range inflation constant				
Dumb form	Uniform (min, max)	0.5–1.0	0.5–1.0	Author’s assumption
Furious form	Uniform (min, max)	1.0–2.0	1.0–2.0	Author’s assumption
**Rabies transmission parameters**				
Duration of the incubation period (days from bite to initial clinical signs)	Lognormal (mu, sigma)	mu = 2.76–3.13 sigma = 0.54–0.75	7.4–65.8	[8,45]
Duration of the pre-clinical infectious period (days from excretion of rabies virus to initial clinical signs)	Gamma (shape, rate)	shape = 0.89–3.79, rate = 0.20–1.16 with a maximum value of 14	0–14	[46]
Duration of clinical infectious period (days from initial clinical signs to death)	PERT (min, ML, max)	0, 3, 12	0–12	[46–50]
Probability of bite if contact				
Pre-clinical or Dumb–within pack	Uniform (min, max)	0.005–0.02	0.005–0.02	Author’s assumption, [40,51,52]
Pre-clinical or Dumb–between packs	Uniform (min, max)	0.1–0.4	0.1–0.4	Author’s assumption, [40,53,54]
Furious form	Uniform (min, max)	0.2–0.8	0.2–0.8	Author’s assumption, [13,49]
Probability of rabies transmission if bite	PERT (min, ML, max)	0.45, 0.49, 0.52	0.45–0.52	[49]
Probability of developing furious form if infected	Uniform (min, max)	0.5–0.75	0.5–0.75	[47,48,55,56]
**Spatio-temporal parameters of first infected case**				
Location of infection	Discrete uniform distribution (1 to 3)	Community Hunter Random	NA	NA
Season of infection	Discrete uniform distribution (1 to 4)	1^st^ day of dry season Midpoint of dry season 1^st^ day of wet season Midpoint of wet season	NA	NA

GSA, Global Sensitivity Analyses; NA, Not applicable; Min, Minimum value; ML, Most likely value; Max, Maximum value.

#### Pack size

We sampled pack sizes from a plausible range of values following a PERT distribution, spanning from 1 to 7 members per pack. The minimal value is consistent with previous reports of lone dingoes in various environmental settings [39,40,54]. The maximal value was based on a mid-range value between 1) the maximum dingo group size observed from camera-traps in the NPA (5 members, [19]), which most likely represents an underestimation of the maximum pack size since members of a pack are not always together at all times and are often found travelling and hunting in sub-groups [40,53] and 2) estimations of dingo pack sizes of up to 9 members on Fraser Island [42,57]. The latter estimate most likely is an overestimation of the maximum pack size in our study area because the Fraser Island population benefits from a resourceful and carefully managed environment. To reflect the seasonal fluctuation of pack sizes due to death or introduction of new young independent dingoes into the group, the assigned PERT distribution mode value was defined within the middle of the range, varying between 3.2 to 4.6 members according to the time at which the primary rabies case occurred (beginning of dry season = 4.1; halfway through the dry season = 3.6; beginning of wet season = 3.2; halfway through the wet season = 4.6). These values were chosen because they generate, on average, a similar number of packs regardless of the season in which the simulation started.

#### Number of days to cover the home range area

No information was found in the literature that indicated the number of days needed for a dingo to cover its home range area or the area covered by a dingo per day. Nevertheless, since the home range sizes reported for the NPA dingo population were relatively small (6.4–33.8 km^2^) and dingoes with similar home range sizes have been found to travel long distances per day (10–20 km/day [43,44]), we assumed that the proportion of home range area traversed by a NPA dingo each day would vary between 20% to 66%, which corresponds to a 1.5 to 5 day period for entire home range coverage. Since it could be expected that a dingo would increase its level of roaming activity in the dry season, either by increasing its speed or the time dedicated for roaming due to a lack of resources and water availability, the number of home range days remained constant in the model for each dingo during all seasons, regardless of the higher home range sizes during the dry season.

#### Variation in home range size and number of home range days due to rabies-induced behavioural changes

Behavioural changes in a rabies-infected dingo might alter the distance the individual roams and the area of land covered per day. To include these rabies-induced behavioural changes into the model, the standard deviation of each infectious dingo’s home range distribution was increased in the case of the furious form and decreased in the case of the dumb form, whilst the number of home range days remained constant. Considering the lack of information in the literature, we assumed that a dingo with the furious or dumb form of rabies would have the radius of its 95% home range area (and hence its standard deviation) increased or decreased by a factor of up to 2, respectively (referred to as “Home range inflation constant”). With these behavioural changes, and considering the ranges of dingo home range sizes in each season, the probability of contact between a clinically infected dingo and a normal dingo would always decrease in the case of the dumb form and, for large distances, increase in the case of the furious form, as illustrated in the example of Fig 2B.

#### Incubation, infectious pre-clinical and infectious clinical period

The incubation period (E) and the pre-clinical infectious period (I1) were based on a lognormal distribution and a gamma distribution, as reproduced by Brookes et al. [8] according to the results from Tojinbara et al. [45] and Fekadu et al. [46], respectively. Since the gamma distribution allows numbers of pre-clinical infectious days up to infinity, a maximum value of 14 days was set for this distribution following results from Fekadu [56], to avoid extreme values for this period (i.e. the model was not allowed to select values >14 from the gamma distribution). In the model, the distribution of latent period was determined by subtracting the distribution of infectious pre-clinical period from the distribution of incubation period, while excluding negative values from the resulting distribution.

Previous studies based on naturally or experimentally infected dogs have reported values ranging between 0 and 12 days [46,50] for the infectious clinical period (I2), with median values of 3 [47] or 4 days [48] and mean values ranging between 3 [49] and 5.7 days [50]. Given the available data in the literature, 3 days was selected as the PERT distribution mode for the infectious clinical period and 0 and 12 days were defined as the minimum and maximum values, respectively; this distribution generates median and mean values of 3.7 and 4.0, respectively, consistent with previous studies.

#### Probability of bite and probability of successful transmission

We assumed that the biting behavior of a dingo infected during the pre-clinical stage (without any clinical signs) or a dingo infected with the dumb form of rabies during the clinical stage would be equivalent to a healthy dingo in a typical context. In normal circumstances, dingo members belonging to the same pack will interact through communal activities in a friendly manner [40]. As reported in a study investigating the behaviour of a pack of captive dingoes, the dominance hierarchy found within a pack could occasionally result in aggression from a dominant dingo to a subordinate, although such behaviours appear to have been rarely observed in the wild [51,52]. The probability of biting for dingoes within a dingo pack is therefore assumed to be low, and most likely slightly lower than what is expected in community dogs (0.7%–7%; [8]), and was therefore estimated to range between 0.5% and 2%. In contrast, encounters between individuals of neighboring packs can lead to aggressive confrontations, involving fighting, as reported by Corbett et al. [53] and Whitehouse [54], as well as Thomson [40] who noted a fighting event in 1 out of 4 observed encounters between individuals of different packs. To reflect this increased level in aggressive behavior, the range of values for the probability of bite given contact between 2 dingoes of different packs was inflated by a factor of 20 with regards to the within-pack probability of bite (0.1–0.4), therefore including the 25% fighting observations from Thomson (40). As dogs exhibiting furious rabies are more likely to bite, we assumed that the behavior of a rabid dingo infected with the furious form during the clinical stage would be twice as aggressive as a healthy dingo meeting a counterpart belonging to another pack. This yields a probability of biting ranging between 0.2–0.8 for a furious rabid dog, consistent with ranges used in a rabies spread model of community dogs [8,49]. Furthermore, we assumed that the biting probability of a dingo exhibiting the furious form would be the same, regardless of whether encounters were with an individual from its own or a different pack.

Finally, we assigned the probability of a successful rabies transmission, given the event of an infectious dingo (I1 or I2) biting a susceptible dingo, according to a PERT distribution, with the mode (0.49), minimum (0.45) and maximum (0.52) values following the results of Hampson, Dushoff (49).

#### Probability of becoming furious

In a large observational study conducted on 957 owned dogs, high proportions (69%) of confirmed rabies-infected dogs were assessed to have the furious form [48]. The same proportion appears to be lower (37%) in an experimental study with 54 rabid dogs [47] and slightly higher (75%) in an observational study conducted on a large sample of 2950 dogs which were mostly categorized as stray [55]. The latter might overestimate the actual proportion of furious dogs, since roaming dogs suffering from the furious form, which exhibit aggressive behavior, are more likely to be reported as suspected cases. To account for the uncertainty related to this parameter, and giving more weight to the results from the study with a large sample size of owned dogs, a uniform distribution of 0.5–0.75 was used to describe the probability that a rabies-infected dingo develops the furious form, as opposed to the dumb form.

### Initiation of infection

At the start of each simulation, the location and time of the year of initiation of infection was determined by first randomly selecting a location scenario and a season scenario, and then by attributing a location and time value according to the selected scenarios.

#### Location scenarios

In the “Community” scenario, it was assumed that the infection was first transmitted from a roaming community dog by assigning the primary case to a dingo from the pack located closest to Injinoo (i.e. the community containing the largest population of roaming domestic dogs [15]). In the “Hunter” scenario, the first infected dingo was assumed to have contracted rabies from a hunting dog in the bush. Thus, the first infection case was assigned to a dingo belonging to a pack located in the area with the highest risk of interaction between hunting dogs and dingoes (according to a risk map in the NPA [20]). In the “Random-location” scenario, the primary case occurred in a pack that was randomly selected across the study area.

#### Season scenarios

The initiation of infection occurred at 4 different times in the year, which were the first day of the dry season (“Dry 1” scenario), the midpoint of the dry season (“Dry 2” scenario), the first day of the wet season (“Wet 1” scenario) and the midpoint of the wet season (“Wet 2” scenario).

### Outcome measures

**Fig 3:**
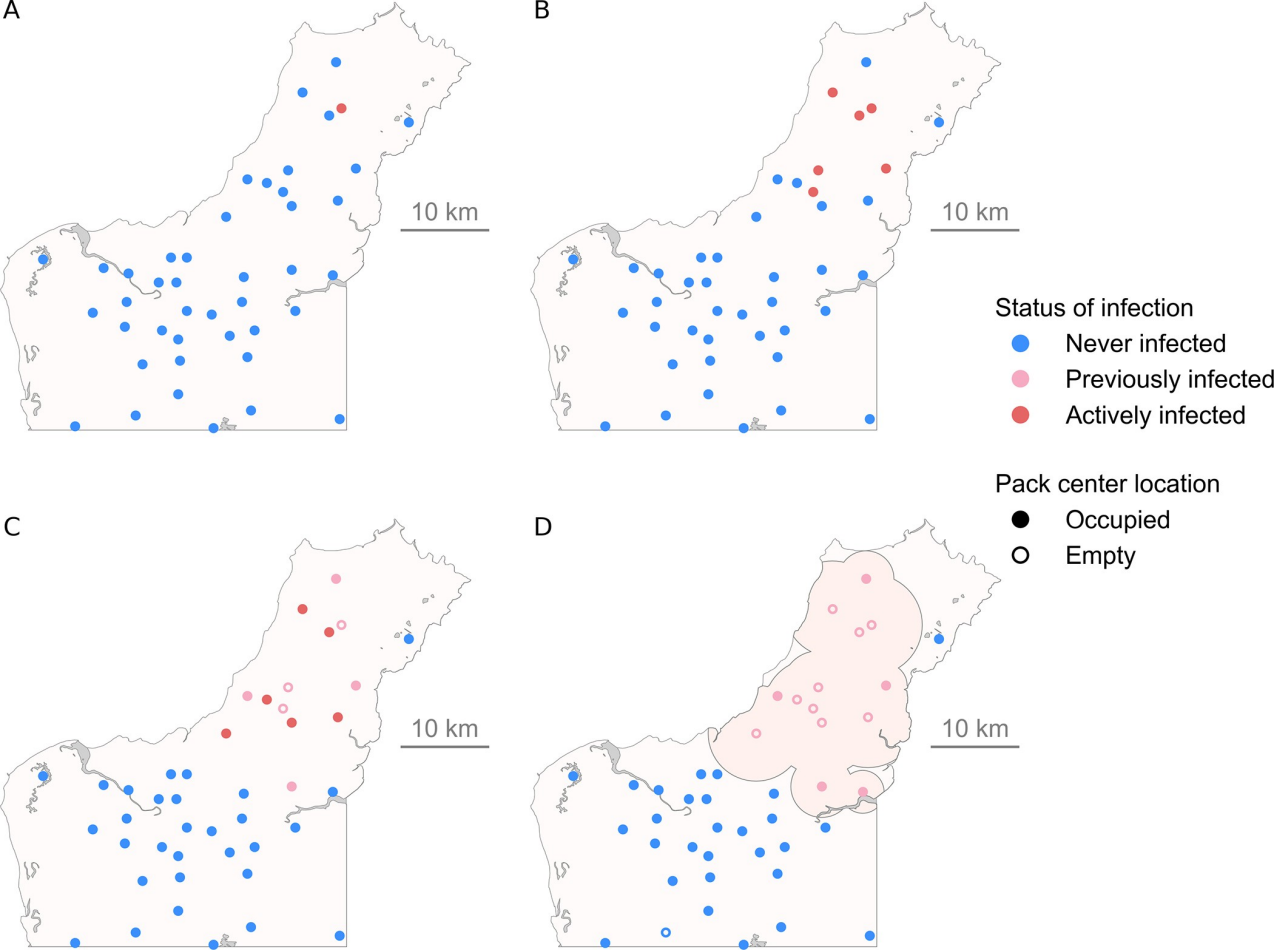
Example of a simulated spatial spread of rabies in a dingo population in northern Australia. Results of a model simulation at time (A) 0, (B) 50 days, (C) 100 days and (D) 167 days (end of the outbreak). The simulation was initiated by the infection of a single primary case (A; red) and terminated once there were no longer any infected dingoes. (A-D) Each dot represents the home range centroid of a pack of dingoes, which can either be categorized as “Never infected”, “Previously infected” (at least one dingo from the pack, whether it has died or moved, was previously infected), “Actively infected” (at least one dingo from the pack is latently infected or infectious). The grey zones represent unsuitable habitat for movement of dingoes during the dry season and the dark-beige zone in (D) represents the area of infection. For this particular simulation, the density of dingoes for the dry season was set at 0.135, the primary case occurred at the beginning of the dry season (Dry 1 scenario) and the “Hunter” scenario was selected for the location of the primary case. All other parameters were randomly chosen, using the specified distributions in Table 1.

For each simulation, the model was run using the parameter values from Table 1 and the following continuous outcome measures were recorded: the total number of infected dingoes (at the end of the simulation, all dingoes that have died of rabies or are currently infected with rabies), the proportion of infected packs (the number of packs that had a least one infected dingo during the simulation over the number of packs at the beginning of the simulation), R_0_ at the individual level (the number of individuals infected by the primary case), R_0_ at the pack level (the number of packs infected by the first infected pack), the duration of the outbreak (the number of days at which no infected dingoes remain), the area of infection (km^2^) and the speed of disease spread (km/week). The area of infection was defined as the area covered, at the end of each simulation, by the 95% home range areas of all dingoes that were infected (by dissolving overlapping areas into one, Fig 3), based on the standard deviation associated with the rabies form (dumb vs furious) in the season when each dingo became infectious. The area covered by unsuitable habitat during the dry season was excluded from the calculation. The dry season was chosen for this calculation because it comprised unsuitable habitats that were inadequate for both seasons. For the speed of disease spread, we measured the wave of disease spread that was created from the incursion point. The average speed of disease spread (km/week) was calculated as the Euclidian distance between the primary case of infection to the furthest point of infection over the entire simulation, divided by the number of weeks between day 0 and the last transmission event.

In addition, two binary outcomes were recorded to assess the probability of spread. Firstly, a binary outcome indicating whether or not the infection was transmitted to at least one pack other than the first infected pack was extracted to evaluate the likelihood of the disease spreading between packs. Also, similar to Reynolds et al. [58], a binary outcome indicating whether or not the disease has spread to at least 10% of the averaged initial population of dingoes was recorded. This proportion was considered to be a threshold at which the risk of transmission to humans or community dogs becomes important. Since the initial population size fluctuates from one season to another and one simulation to another, the 10% proportion was calculated by using the averaged initial population size based on the total number of simulations ran for output presentation (200,000 simulations, see section *Summary statistics*), so as to obtain comparable numbers from one simulation to another.

### Convergence testing

The number of simulations needed to achieve convergence of summary statistics was calculated using a method described by Brookes et al. [8]. Briefly, the nine outcome measures from the model were recorded for 200,000 simulations. For each outcome measure, an increasing number of outputs (from 1 to 20,000, referred to as “size of simulation set”) were re-sampled randomly from the outputs of the 200,000 simulations. For the continuous outcome measures, only simulations for which the infection has spread to at least one other pack were sampled. This random sampling process was repeated 10 times, resulting in 10 sets of outputs for each size of simulation set (for example, the size of simulation set 200 included 10 sets of outputs, each composed of 200 simulations, the size of simulation set 201 included 10 sets of outputs, each composed of 201 simulations, and so on). The median for continuous outcome measures and the mean for binary outcome measures were calculated for each set of outputs. For each size of simulation set, the coefficient of variation, based on the 10 median/mean outputs of a given outcome measure, was computed. We considered that convergence was achieved for an outcome measure when the coefficient of variation of a size of simulation set was lower than 2.5% for all 100 previous sizes of simulation sets. The location and season scenarios were randomly selected for each of the 200,000 simulations and the convergence testing was performed for all simulations, regardless of scenarios, because this generated the most variance in model outputs. Results of the convergence testing were used to evaluate if 200,000 simulations generated a sufficient number of simulations to report statistical outputs for each scenario.

### Summary statistics

Summary statistics of the outcome measures were used to describe the probability of outbreak and its extent. Scenario outcomes were compared by grouping the outputs of simulations from each of the seven scenarios. These summary statistics included the median value for the seven continuous outcome measures, the mean value for the two binary outcome measures (the proportion of simulations in which each outcome was present) and the range between the 2.5^th^ and 97.5^th^ percentiles for all nine outcome measures distributions. The summary statistics were derived from all 200,000 simulations for the two binary outcome measures and from the subset of simulations in which rabies has spread to at least one other pack for the seven continuous outcome measures.

### Global sensitivity analyses

A Sobol’ sensitivity analysis, which describes the contribution of each parameter on the variance of each respective model outcome, was performed on all 14 input parameters from the model, excluding the categorical spatio-temporal parameters on initiation of infection, using the SALib module in python [59]. A random number generator seed was also treated as an additional parameter to absorb the variance caused by the stochasticity of the model [27]. Consequently, at the beginning of each simulation which was carried out for this exercise, a seed value was randomly sampled from a uniform distribution (0–100). A total of 136,000 sets of parameters were generated by employing Sobol’ sequences, according to a specific range of values for each of the 14 input parameters (Table 1) and the additional random seed number variable. The model was run 136,000 times using each set of parameters, and these parameters were assigned to all events or individuals and remained fixed throughout each given simulation, regardless of season. Therefore, although the home range size parameter in the present model changed at every half-season as the simulation progressed, for the purpose of sensitivity analysis, the selected value remained fixed throughout the duration of each simulation. All scenarios were considered in the analyses by randomly selecting the season and the geographic location of the primary case.

Based on the outputs from all 136,000 simulations, the first-order (individual effect of the parameter to the output variance) and total order (total effect of the parameter to the output variance, considering the individual effect of the parameter and its interactions with all other parameters of the model) Sobol’ sensitivity indices and their corresponding 95% confidence intervals were calculated for each outcome-input parameter combination (9 × 15 combinations).

### Relationship between outcome measure and input parameter

Using the outputs from the 136,000 simulations of the Sobol’ sensitivity analyses, data from each outcome measure was grouped into bins of equal interval, based on the range of values sampled for each input parameter. The median value (continuous outcome measures) or the mean value (binary outcome measures) of outputs was calculated for every bin of every input parameter. The resulting data was plotted, thus illustrating the relationship of each outcome-input variable combination (i.e. 9 × 15 combinations).

## Results

### General model outputs

**Fig 4:**
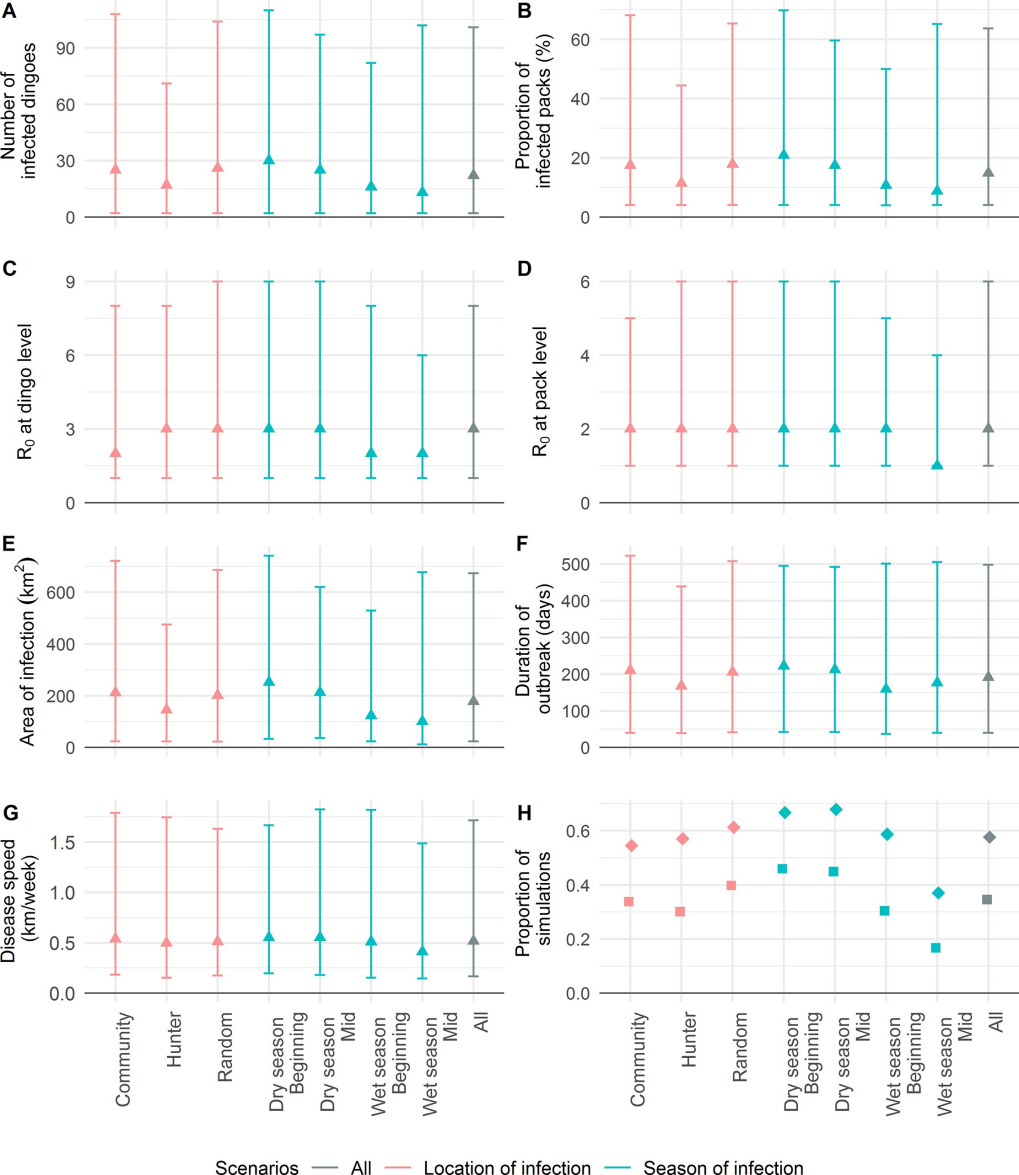
Outputs obtained from 200,000 simulations of a spatial rabies spread model within a dingo population. Outcome measures include (A) number of infected dingoes, (B) proportion of infected packs, (C) R_0_ at the dingo level, (D) R_0_ at the pack level, (E) area of infection, (F) maximum distance of infection, (G) speed of disease spread and (H) proportion of simulations for which infection has spread to more than one pack and more than 10% of the initial dingo population. For (A-G), the distribution of outputs reported are based on simulations in which disease has spread to at least one other pack, whereas proportions in (H) are based on all simulations. Triangles represent median values, diamonds represent the proportion of simulations with spread of disease to more than one pack and squares represent the proportion of simulations with spread of disease to at least 10% of the averaged initial dingo population. The vertical bars correspond to the range between the 2.5^th^ and 97.5^th^ percentiles of values distribution.

Overall, considering the 200,000 simulations, a canine rabies outbreak involving more than one dingo pack occurred in 58% of simulations (Fig 4). At least 16 dingoes were infected (10% of the average initial population of 159 dingoes) in 35% of simulations. Seventy-six percent and 77% of simulations in which infection spread to >1 pack and >10% of the dingo population, respectively, were initiated by a rabid dingo which developed the furious form of rabies. In contrast, when considering simulations that did not lead to the spread of rabies in >1 pack and >10% of the dingo population, this proportion dropped to 45% and 55%, respectively. In simulations in which > 1 pack was infected, rabies spread at a median speed of 0.52 km/week, infecting a median of 22 dingoes (14% of the average initial population) and 15% of packs, covering a median area of 177 km^2^ over a median of 191 days. The median value of R_0_ at the dingo level was 3, higher than the median value of 2 at the pack level. Almost all simulations eventually died out (i.e. 97.5% percentile of outbreak duration was equal to 498 days for simulations in which disease spread to at least one other pack). One simulation terminated at the limit of 3 years (149 dingoes infected). Disease spread persisted for >1 year and > 2 years in 6% of simulations (n = 12,190) and 0.1% (n = 181) of simulations, respectively.

### Scenario model outputs

The number of simulations needed to achieve convergence varied between 268 for R_0_ at the pack level to 11,600 for R_0_ at the dingo level (S1 and S2 Figs). Therefore, all scenarios investigated, including the group with the lowest number of simulations (i.e. 18,414 simulations in scenario Wet 2 considering only simulations with more than one pack infected), contained enough simulations for convergence of all outcome measures. Predicted outputs for each outcome measure and each scenario are illustrated in Fig 4 and detailed in S1 Table.

For the location scenarios, the proportion of simulations with at least 10% of the population infected and the median outputs of all continuous outcome measures, except for R_0_ at the dingo and pack levels, were lowest for the hunter scenario. The community and the random-location scenarios displayed greater values, which were comparable to one another. The median values for R_0_ at the pack level were equal between all three scenarios. In contrast to all other outcome measures, R_0_ at the dingo level and the proportion of simulations with spread to another pack were lowest for the community scenario.

Regarding the seasonal group scenarios, all outcome measures were overall higher in the dry seasons compared to the wet seasons. More specifically, the proportion of simulations with >10% dingoes infected and the median values for the number of dingoes infected, the proportion of infected packs, the area of infection and the disease speed were highest for the Dry 1 season and decreased in the order of seasons to reach their lowest values in Wet 2 season. Exceptions to this pattern were that Wet 2 scenario resulted in a slightly higher median value of duration of outbreak than Wet 1 scenario and the proportion of simulations with >1 infected pack was slightly higher for Dry 2 scenario compared to Dry 1 scenario. The median values of R_0_ at the dingo level in the dry season (3) were higher than the wet season (2), and the median values of R_0_ at the pack level were equal between all scenarios (2), except for the Wet 2 season in which R_0_ was lower (1).

### Global sensitivity analyses

**Fig 5:**
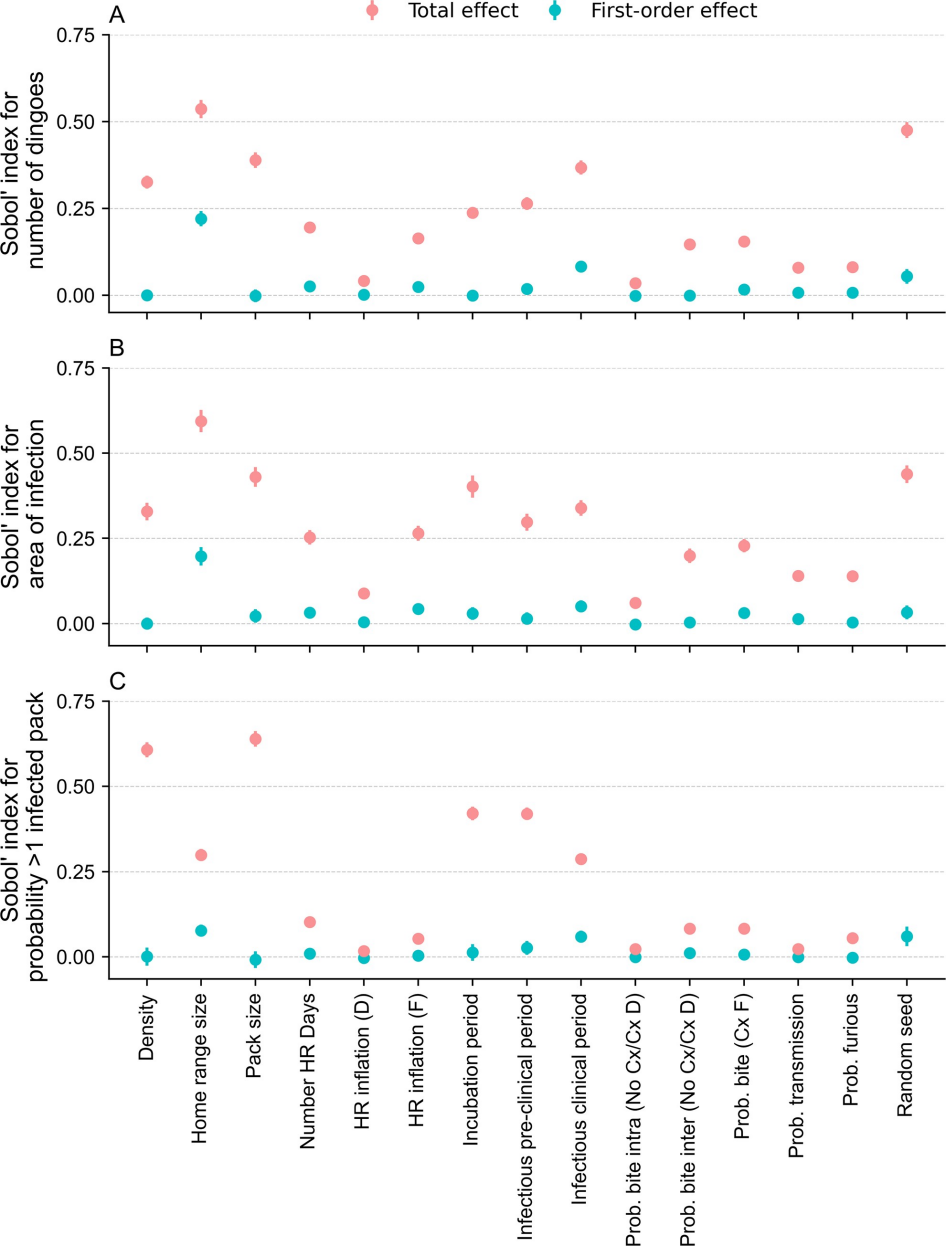
Global sensitivity indices for input parameters of a rabies spread model within a dingo population. First order (cyan) and total effect (pink) Sobol’ sensitivity indices for each input parameter on (A) the number of infected dingoes, (B) the area of infection and (C) the binary outcome indicating whether the infection has spread to at least one other pack, following the incursion of rabies within the dingo population. Error bars represent the 95% confidence interval for each Sobol’ index. The abbreviation “Prob.” stands for “probability”, “HR” for “Home range”, “Cx” for “clinical”, “D” for “dumb form” and “F” for “furious form”.

The first-order and total effect Sobol’ indices of all model input parameters are illustrated in Fig 5 for the number of infected dingoes, the area of infection and the binary outcome indicating whether or not rabies spread to at least one other pack. These were considered to be the three model outcomes which best measured the level of risk for public health, the spatial spread of disease and the probability of spreading to packs. Sobol’ indices for all other outcome measures are shown in S3 and S4 Figs.

In the first-order Sobol’ sensitivity analyses, for all outcome measures except for R_0_ at the dingo level, disease speed and duration of outbreak, home range size and infectious clinical period were the first and the second most influential input parameters of the model, each accounting for 8–23% and 5–8% of the model output variability, respectively. The infectious clinical period was responsible for most of the variance for R_0_ at the dingo level (11%) whereas the most dominant parameter for disease speed and duration of outbreak was the incubation period (28% and 18%, respectively), followed by home range size as the second most influential parameter for all three outcome measures (6–10%). All other parameters, except for infectious clinical period in the case of duration of outbreak (7%), contributed less than 5% of model output variability, indicating that these parameters were not significantly influential on their own [60].

The sensitivity indices for most parameters were drastically increased when input interactions were taken into account in the total effect analyses, therefore indicating that these parameters strongly interact with each other, and these interactions contribute significantly to the variability of model outputs. Most model input parameters were influential, with total-order sensitivity indices above 5%. Several parameters did not contribute significantly to model variance for most outcome measures, including the home range inflation constant for the dumb form of rabies and the probability of bite for a dingo (No clinical signs/Dumb form) contacting a dingo from the same pack. For each outcome measure, the density, home range size and pack size were found to be the three most dominant ecological parameters of dingoes in the model, whereas the incubation, the infectious pre-clinical and the infectious clinical periods corresponded to the most dominant rabies transmission parameters, each possessing a total-order sensitivity index above 24%, except for R_0_ at the dingo level. For the latter outcome measure, density and pack size had greatest influence, explaining more than 50% of the variance.

### Relationship between outcome measures and input parameters

The plots illustrating each outcome measure as a function of each input variable in the model (S5–S13 Figs) reveal a positive monotonic relationship of different level for most outcome-variable combinations. As an exception, the number of home range days is inversely related to all outcome measures. Similarly, an increase in pack size results in a decrease in the proportion of simulations with more than one infected pack and the speed of disease spread. In addition to these two outcomes measures, the incubation period is also inversely related to the area of infection. Consistent with the findings from the first-order Sobol’ sensitivity analyses, we observe that the infectious clinical period is strongly related to R_0_ at the dingo level, the incubation period results in a significant decrease and increase in disease speed and duration of outbreak, respectively, and the home range size causes a drastic increase in all other model outputs. A horizontal trend line is noticed in the random seed number parameter for each outcome measure, indicating that model outputs are independent of the selection of the random seed number, as expected.

## Discussion

Our study is the first to model the spatial spread of rabies within the dingo population of northern Australia based on robust field-derived ecological parameters, including movement and density of the NPA dingo population. Furthermore, the incorporation of rabies-induced behavioural changes and seasonal fluctuations in ecological processes, including landscape barriers, dingo births, and home range sizes, provides a realistic representation of the potential wildlife-mediated rabies-dingo system in our study area. The key predictions of our model include the high likelihood that one single infected dingo would lead to the spread of rabies in the population, with the potential of outbreaks of considerable size (in terms of number of dingoes infected, spatial coverage and duration), therefore posing a substantial risk for public health.

According to our model, we estimate that there is a 58% probability of rabies spreading between dingo packs and a 35% probability of an outbreak of significant size occurring when the disease is introduced into a susceptible dingo population in the NPA. However, despite the incorporation of population turnover in the model, including the introduction of young susceptible dingoes into the population and dispersal of individuals in areas which could be surrounded by infected packs, our results suggest a high probability that outbreaks are self-limiting within a year (~94%). Hence, in contrast to the findings from Johnstone-Robertson, et al. [27], our model indicates that the establishment of a sustained rabies infection is unlikely in the NPA dingo population. A mixture of ecological characteristics specific to our target population might explain this frequent disease die-off. Our previous field studies have shown that dingo density and home range size in the NPA are in the lower range of values reported in the literature, consistent with estimates from other Australian subtropical and tropical environments [17,61]. As demonstrated in the global sensitivity analyses of our model, both parameters have a strong influence on spread of disease. Along with the low NPA dingo density estimates, individuals from our model were further grouped into packs, resulting in a sparse spatial distribution of packs across the study area, interrupted by landscape features including the ocean coast and various waterbodies. Despite the high rate of rabies transmission between members of the same pack—as evidenced by the large median value of R_0_ at the dingo level—the daily probability of contact between individuals of neighboring packs declines sharply as distance between packs increases and as home range sizes decrease, thus facilitating disease die-out.

On a similar note, the median speed of propagation of the epizootic front in the present study (i.e. 0.52 km/week) is less than half that predicted from another rabies spread model [27] (i.e. 67 km/year, which corresponds to 1.3 km/week). In addition to differences in parameterisation as discussed above, this dissimilarity could also be attributed to different calculation approaches in disease spread. In the latter study, the epidemic speed was calculated for simulations in which the disease has percolated beyond a distance of 120 km, as opposed to our study in which the propagation speed was calculated from all simulations in which the disease has spread beyond one infected pack, including outbreaks of smaller magnitude as well as larger outbreaks. Our results are nonetheless consistent with, albeit at the lower end of the range, field data from rabies infection in domestic dogs (0.5 km/week) [62] and foxes (0.57–1.15 km/week) and simulation results from rabies spread models in foxes (0.8 km/week) [63].

The scenario in which a dingo contracts rabies from a domestic dog roaming freely around the communities, as opposed to the hunter scenario, leads to the largest outbreak sizes in terms of space, time and population infected. Furthermore, this scenario implicitly involves a higher risk for public health, due to the proximity of the initial spread of rabies in the dingo population and members of the communities. Concomitantly, the chances of early detection of the outbreak are increased due to proximity of this area to human populations, and the spread of rabies might consequently be easier to contain if timely control measures (such as vaccination) are established in the dingo population. The smaller outbreak outcomes in the hunter scenario could be explained by the fact that the areas with the highest risk of interaction between hunting dogs and dingoes are located towards the tip of the peninsula (Fig 3). In this scenario, the spread of rabies is limited to the south only, compared to other potential incursion points in the study area which allow multi-directional spread. Compared to other scenarios, the slightly lower proportion of simulations with >1 infected pack in the community scenario might be the reflection of the close proximity of the primary case to the ocean coast, thus reducing the likelihood of a successful transmission to another pack. However, compared to the hunter scenario, once this first inter-pack transmission is achieved, the magnitude of the outbreak is expected to be larger.

As anticipated, the likelihood of rabies successfully spreading beyond the first infected pack and the corresponding spatial extent of the outbreak tends to be greater when rabies is introduced during the dry season. In fact, the larger amount of area traversed per day during the dry season (greater home range size relative to the number of home range days), reflecting the necessity for dingoes to intensify their level of roaming activity over large distances due to the scarcity of food and water resource availability in the dry season, will increase the probability of contact of dingoes between neighboring packs, resulting in larger outbreaks and larger areas infected. This is consistent with Sparkes et al. [11] who proposed that dingoes from resource poor areas, which usually occupy home ranges of greater extent, may be responsible for the spread of rabies over a larger land area. Furthermore, spatial heterogeneity is more extensive in the wet season, as the habitat is composed of a greater proportion of natural barriers to dingo movement, consequently reducing the probability of contact between packs. Various modelling studies have revealed that natural landscape features, such as rivers, can act as an effective barrier to animal movement and consequently impede rabies flow [23,64], consistent with the findings of our model.

The three ecological parameters with the greatest influence on model outputs are based on data either estimated directly from the field (i.e. density and home range size) or derived from observations of the dingo population in our study area (i.e. pack size). The variance in model outcomes resulting from these parameters is expected to reflect the uncertainty in our estimates measured from the field as well as seasonal and individual fluctuations in the natural processes of our study area, rather than uncertainty resulting from the use of a large range of plausible values due to knowledge gaps in the literature. However, the variance of the home range size may have been underestimated since the range used for this parameter in the model was based on the 95% confidence interval from the mean home range size estimate of the population, as opposed to individual variability. Consistent with the global sensitivity analysis findings from other rabies spread simulations in domestic dogs [8], the incubation and clinical periods strongly influence model outcomes in our model. Once again, these parameters have been documented extensively through field and experimental studies and have been proven to vary greatly at an individual level. In contrast, data in the literature regarding the preclinical infectious period is limited. Given the sensitivity of model outputs to this parameter, further research in this area would help improve rabies spread model predictions. Although not ranked as the most influential variables, rabies spread parameters related to the furious form of rabies are shown to have a significant impact on most outcome measures. These results, which are based on the model’s assumption, support the hypothesis that furious dogs considerably affect rabies outbreaks, and are consistent with the findings from a previous modelling study which evaluated the influence of rabies-induced behavioural parameters on predicted rabies outbreaks in domestic dogs [8]. Other rabies spread parameters which denoted high uncertainty due to the lack of available information in the literature, notably the home range inflation constant for the dumb form of rabies and the probability of bite for healthy or dumb-infected individuals, are not found to be influential.

The evaluation of the input-outcome relationships allowed the improved understanding of the behavior of each parameter in the model. From this analysis, two unanticipated findings were identified regarding the incubation period and pack size parameters. First, as expected, an increase in the incubation period resulted in the duration of outbreaks to lengthen and the speed of disease spread to diminish, since the time between transmission events would increase accordingly. On the other hand, the incubation period’s slight negative relationship with the area of infection and the probability of disease spread to >1 pack is not as straightforward. Considering that approximately 21% of the dingo population dies of natural cause within a year in the model, a possible explanation is that when the incubation period increases, the likelihood of a latently infected dingo dying of a natural cause before reaching the infectious pre-clinical stage also increases, thus preventing the transmission of rabies. Second, the positive influence of the pack size that was found on the number of infected dingoes, the proportion of infected packs, R_0_ at the dingo level and the proportion of simulations with infection of >10% of the dingo population can be explained by an increased number of dingoes that one rabid individual can potentially infect within its pack. However, the pack size also has a direct impact on the spatial distribution of packs; as the number of dingoes within each pack expands, the total number of packs to reach the initial population estimate within the study area diminishes, resulting in a sparser distribution of packs. Contact between members of different packs is thus inhibited, which may explain the observed lower disease speed and probability of infecting >1 pack as pack size increases.

The disease spread dynamics in our model were fundamentally based on the concept of space sharing between individuals [65]. The application of a bivariate normal function allowed us to define the probability density of an animal’s use of space, similar to an animal’s utilization distribution [66], leading to a relatively simple and, to our knowledge, novel equation for estimating the probability of contact between individuals (Eq 4). This formula could be applied to other animal populations in which utilization distribution estimates are unavailable, yet population densities and home range sizes are known. One important caveat with our probability of contact approach is our assumption of a circular home range distribution, meaning that the probability distribution is the same in all directions. The home range distributions of dingoes in the NPA are most likely a complex function involving multiple spatial attributes in the study area such as resource focal points, landscape barriers, and corridor pathways used by dingoes to travel through the dense vegetation. However, considering the lack of evidence supporting a specific home range shape in our study area, we believe that this assumption represents a valid approximation of the actual dingo movement in the NPA. The use of GPS collars on the NPA dingo population could provide precise information on the shape of the home range distributions and the variability in home range size between individuals.

The clustering of dingoes into pack entities allowed assessment of contact at 2 levels. Differences in the intra and inter-pack contact probabilities were consistent with the close social behaviors for members of a same pack as opposed to the low frequency of observed interactions of individuals from different packs in the wild [39,52–54,67]. Contact heterogeneity between members of different packs was incorporated in part by the propensity of each individual to roam around its home range centroid point (determined by the home range size and number of home range days). However, dingoes were assumed to roam randomly within their respective home range distribution and independently to dingoes from other packs, which might not always be a valid assumption because dingoes might exhibit a large range of social behaviors ranging from active avoidance to attraction during the mating period. The incorporation of a more realistic representation of the heterogeneous social behavior inherent to dingoes, which most likely varies according to breeding seasons and individual characteristics such as sex and age, could contribute to improved model outcome predictions. However, dingo contact behaviour represents a major research gap in the literature, especially in tropical and equatorial ecosystems of northern Australia where data is non-existent [17], and highlights the need for future research in this field.

It is important to note that the present model was restricted to a small area to avoid extrapolation of our data to a population with differing ecological characteristics. Thus, although our model indicates that the ecological characteristics in the NPA will most likely not support a sustained infection within the dingo population, we did not evaluate whether rabies spread would be likely to stop naturally or continue its path southward, infecting contiguous dingo populations in Queensland. Furthermore, the particular context of our study area, with consistent opportunities for interactions between NPA community dogs and dingoes, could facilitate the development of overlapping domestic dog-mediated and wildlife-mediated rabies cycles, which may be sufficient to allow for endemicity. Considering the proximity of Indigenous community members to dingo habitat and increasing evidence of the presence of dingoes in and around the NPA communities [19,20], the potential of a large and long-lasting rabies outbreak in the NPA dingo population represents a significant risk for rabies transmission to humans. The findings of this study therefore highlight the importance of preventing the introduction of rabies into the NPA by implementing effective surveillance and prevention measures within this high-risk area for rabies incursion.

Two main strategic directions in terms of control interventions can be considered in light of the main findings of the present study. Firstly, in the event that rabies is detected within the NPA dingo population or the domestic dogs in the communities, control strategies should prioritize vaccination of the domestic dogs as they represent a direct threat to humans. Moreover, vaccination programs involving distribution of oral vaccine baits targeting dingoes, a commonly used and effective technique to control rabies spread in wild carnivores [68–70], could strategically target zones around the communities as a priority, considering that infection in this area is associated with larger outbreaks leading to a greater spatial spread. This approach could also assist in preventing an incursion or re-incursion of rabies into the dingo population from a clinically or sub-clinically infectious free-roaming dog, as well as providing barriers around the communities by reducing the risk of rabies transmission from infectious dingoes to susceptible community dogs and, ultimately, to humans. To test this hypothesis, various control measures which might be implemented in the NPA dingo population in the face of a rabies outbreak, including spatially strategic control programs that would accommodate for heterogeneity in landscape features [71,72], could be further explored using the present rabies spread model.

## Supporting information

S1 Appendix Details of calculations for the probability of death, the probability of birth, the probability of relocation and the probability of contact, and details on the network created to simulate the movement of dingoes in a spatial rabies spread model in the dingo population of the Northern Peninsula Area of Queensland, Australia. (PDF)

S1 Table Summary statistics (median, 2.5th percentile and 97.5th percentile) for each scenario when considering the subset of simulations which resulted in more than one pack infected and when considering all 200,000 simulations. (PDF)

S1 Fig Plots of the coefficient of variation of 10 sets of output simulations against the size of simulation set. The size of simulation set represents the increasing number of simulations (x axis) in each of the 10 sets of output simulations. The outputs include (A) the number of infected dingo, (B) the proportion of infected packs, (C) R_0_ at the dingo level, (D) R_0_ at the pack level, (D) the area of infection, (E) the duration of outbreak, (F) the speed of disease spread, (G) the binary outcome indicating whether or not the infection has spread to more than one pack and (H) the binary outcome indicating whether or not the infection has spread to at least 10% of the initial population. The horizontal red line represents a coefficient of variation at 2.5%.
(TIF)

S2 Fig Proportion of sizes of simulation sets, based on the previous 100 sizes of simulation sets, which had a coefficient of variation of < 2.5%. The outputs include (A) the number of infected dingo, (B) the proportion of infected packs, (C) R_0_ at the dingo level, (D) R_0_ at the pack level, (D) the area of infection, (E) the duration of outbreak, (F) the speed of disease spread, (G) the binary outcome indicating whether or not the infection has spread to more than one pack and (H) the binary outcome indicating whether or not the infection has spread to at least 10% of the initial population.
(TIF)

S3 Fig **Global sensitivity indices for input parameters of a rabies spread model within a dingo population on (A) the proportion of infected packs, (B) R**_**0**_ **at the dingo level and (C) R**_**0**_ **at the pack level.** The cyan and pink symbols represent the first order and total effect Sobol’ sensitivity indices. Error bars represent the 95% confidence interval for each Sobol’ index. The abbreviation “Prob.” stands for “probability”, “HR” for “Home range”, “Cx” for “clinical”, “D” for “dumb form” and “F” for “furious form”.
(TIF)

S4 Fig **Global sensitivity indices for input parameters of a rabies spread model within a dingo population on (A) the speed of disease spread, (B) the duration of outbreak and (C) the binary outcome indicating whether or not the infection has spread to at least 10% of the initial population.** The cyan and pink symbols represent the first order and total effect Sobol’ sensitivity indices. Error bars represent the 95% confidence interval for each Sobol’ index. The abbreviation “Prob.” stands for “probability”, “HR” for “Home range”, “Cx” for “clinical”, “D” for “dumb form” and “F” for “furious form”.
(TIF)

S5 Fig Plot of the relationship between the number of infected dingoes against all 14 input parameters of the model and the random seed number. The input parameters include: Density, home range size, pack size, number of home range days, home range inflation constant for dumb infected dingoes, home range inflation constant for furious infected dingoes, incubation period, infectious pre-clinical period, infectious clinical period, probability that a non-clinical or dumb infected dingo bites another member of the pack, probability that a non-clinical or dumb infected dingo bites a dingo from another pack, probability of bite for a furious infected dingo, probability of rabies transmission given the event of a bite, probability of developing the furious form of rabies.
(TIF)

S6 Fig Plot of the relationship between the proportion of infected packs against all 14 input parameters of the model and the random seed number. (See legend of S5 Fig).
(TIF)

S7 Fig Plot of the relationship between R_0_ at the dingo level against all 14 input parameters of the model and the random seed number. (See legend of S5 Fig).
(TIF)

S8 Fig Plot of the relationship between R_0_ at the pack level against all 14 input parameters of the model and the random seed number. (See legend of S5 Fig)
(TIF)

S9 Fig Plot of the relationship between the area of infection against all 14 input parameters of the model and the random seed number. (See legend of S5 Fig).
(TIF)

S10 Fig Plot of the relationship between the duration of outbreak against all 14 input parameters of the model and the random seed number. (See legend of S5 Fig).
(TIF)

S11 Fig Plot of the relationship between the speed of the disease spread against all 14 input parameters of the model and the random seed number. (See legend of S5 Fig).
(TIF)

S12 Fig Plot of the relationship between the proportion of simulations with more than one pack infected against all 14 input parameters of the model and the random seed number. (See legend of S5 Fig).
(TIF)

S13 Fig Plot of the relationship between the proportion of simulations with more than 10% of the population infected against all 14 input parameters of the model and the random seed number. (See legend of S5 Fig).
(TIF)
