# Supplementary material for: Could a rabies incursion spread in the northern Australian dingo population? Development of a spatial stochastic simulation model
Source: PLoS Negl Trop Dis. 2021 Feb 12;15(2):e0009124. doi: 10.1371/journal.pntd.0009124 (PMC7906478; doi:10.1371/journal.pntd.0009124)
Supplement: S1 Appendix — (PDF) [file pntd.0009124.s001.pdf]

Appendix

Could a rabies incursion spread in the northern Australian dingo population? Development of a spatial stochastic simulation model

This document contains all calculations in relation to the probability of dying, probability of dingo introduction, home range distribution, probability of relocation and probability of contact as well as details on the network created to simulate the movement of dingoes from a rabies spread model in dingo populations from the NPA. In this document, all formulas in blue were used directly in the model.

Table A. List of variables from all equations used in a spatial model to simulate the rabies spread in the dingo population from the Northern Peninsula Area of Queensland.

Parameter symbol	Parameter name	Value* (unit)
N	Number of days in a year	365 (days)
k	Number of days during the dry season	185 (days)
s	Number of days during the wet season	180 (days)
b	Number of days during the period of introduction of new independent dingoes	90 (days)
A	Area of the study region	1131 (km^2)
δ	Death rate	NA
PD_{daily}	Daily probability of a dingo dying	NA
C	Value of the daily probability of dingo introduction in the population following new births, during the first half of the wet season	NA
$\mathrm{Mean}_{\mathrm{D\_dry}}$	Mean density during the dry season	(dog/km^2)
$\mathrm{Mean}_{\mathrm{D\_wet}}$	Mean density during the wet season	(dog/km^2)
P_D	Population size at the beginning of the dry season	(dogs)
$P_{D\_0.5}$	Population size at the midpoint of the dry season	(dogs)
P_W	Population size at the beginning of the wet season	(dogs)
P_b	Population size at the end of the period of introduction of new dingoes (which is approximately the midpoint of the wet season)	(dogs)
$P_{D'}$	Population size at the end of the dry season (which is equal to P_W)	(dogs)
$P_{W'}$	Population size at the end of the wet season (which is equal to P_D)	(dogs)
R_{95}	Radius for the 95% home range size of a dingo	(km)
σ_i	Standard deviation of the home range distribution of dingo *i*	(km)
d	Distance between two home range centroid points	(km)
n_i	Number of days needed for dingo *i* to explore most of its home range area	(days)
$P_i(x,y)$	Home range distribution for dingo *i*	
PR_{i_daily}	Daily probability of dingo *i* relocating at a new centroid point of distance *d*	NA
PC_{ij_daily}	Daily probability of contact between dingo *i* and dingo *j*	NA

*Values are given when the variable is a constant
NA = Not applicable (no corresponding value or unit)

Probability of dying and probability of introduction of newly born dingoes

The study area, in northern Australia, is characterised by an equatorial climate, with a dry season (approximately May-October) and a wet season (approximately November-April). According to the camera-trap study results, the dingo density varies between the dry season (0.135 dingoes/ km^2 (CI: $0.127 - 0.144$)) and the wet season (0.147 dingoes/ km^2 (CI: $0.135 - 0.159$)). These values are interpreted as the mean density throughout each season (~6 months each). This temporal fluctuation of the population is incorporated into the rabies spread model by natural death and birth:

1. The daily probability of dying for each dingo is described by

$$PD_{i_{daily}} = 1 - e^{-\delta} \quad (1)$$

   where δ is the death rate. It is assumed that the death rate is constant across all seasons (but varies between simulations).

2. The probability of introduction of new independent dingoes into the population occurs during a specific time period of the year, that is during the first half of the wet season. During this period of dingo introduction, the probability of introduction is set to be equal to a simple constant, as illustrated below:

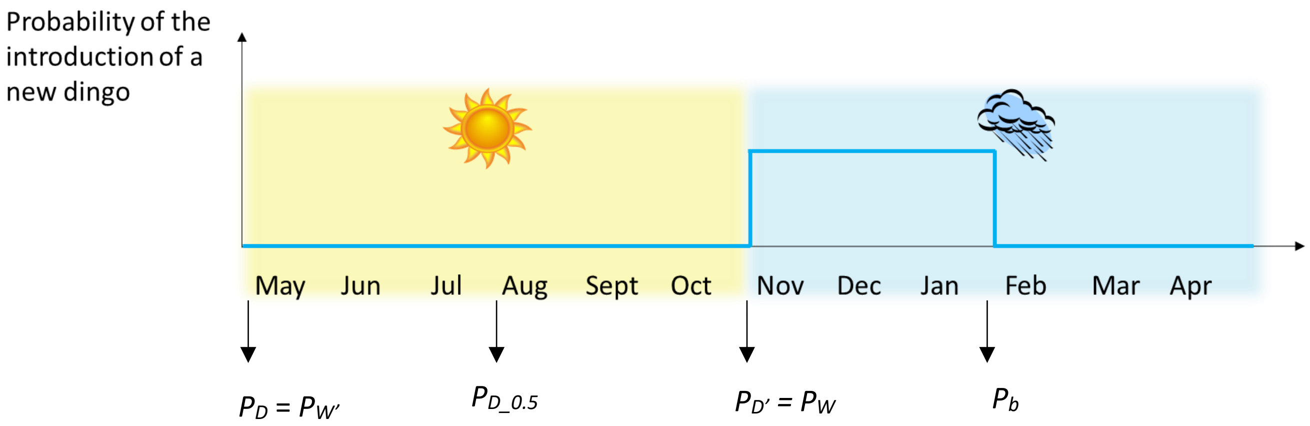

Figure A. Probability of introduction of newly born dingoes throughout the year (blue line), during the dry and wet seasons, in the NPA. P_D , $P_{D\_0.5}$, $P_{D'}$, $P_{W'}$, P_b and $P_{W'}$ stand for the population size at different times in the year (i.e. beginning and midpoint of the dry and wet seasons). During the first part of the wet season, the probability of introduction of a new dingo is equal to C.

It is assumed that the population size is stable from one year to another.
The number of days during the dry season is k, during the wet season is s and during the period of introduction is b. On day 0 of the dry season, the population size is P_D. At the end of the dry season, on day k, the population size is $P_{D'}$, which is also equal to the population size at the beginning of the wet season P_W (day 0 of wet season). The population size at the midpoint of the dry season is $P_{D\_0.5}$. At the end of the wet season, on day s, the population size is $P_{W'}$, which is also equal to the population size at the beginning of the dry season P_D. The population size at the end of the birth period is P_b, which corresponds approximately to the midpoint of the wet season.

DRY SEASON

On day 1, the population size is:

$$P_1 = P_D - P_D(1 - e^{-\delta})$$
$$P_1 = P_D e^{-\delta}$$

On day 2, the population size is:

$$P_2 = P_1 e^{-\delta}$$
$$P_2 = P_D e^{-2\delta}$$

By recursion, on day n, the population size is:

$$P_n = P_D e^{-n\delta} \tag{2}$$

The average daily density during the dry season must equal:

$$Mean_{D\_dry} = \frac{P_1 + P_2 + P_3 + \ldots + P_{D'}}{kA}$$

$$Mean_{D\_dry} = \frac{1}{kA} \sum_{n=1}^{k} P_n$$

$$Mean_{D\_dry} = \frac{P_D}{kA} \sum_{n=1}^{k} e^{-n\delta}$$

$$P_D = \frac{kA \times Mean_{D\_dry}}{\sum_{n=1}^{k} e^{-n\delta}}$$

The geometric series sums to:

$$\sum_{n=1}^{k} e^{-n\delta} = \frac{e^{-\delta} - e^{-(k+1)\delta}}{1 - e^{-\delta}} \tag{3}$$

This yields the population size at day 0, at the beginning of the dry season.

$$P_D = \frac{kA \times Mean_{D\_dry}(1 - e^{-\delta})}{e^{-\lambda} - e^{-(k+1)\delta}} \tag{4}$$

Using equation (2) and (4), we can calculate the population size at the midpoint of the dry season, on day *k/2*

$$P_{D\_0.5} = \frac{kA \times Mean_{D\_dry}(1 - e^{-\delta})}{e^{-\lambda} - e^{-(k+1)\delta}} e^{-k\delta/2} \tag{5}$$

Similarly, the approximate population size on the last day of the dry season, on day *k*, becomes:

$$P_{D'} = \frac{kA \times Mean_{D\_dry}(1 - e^{-\delta})}{e^{-\delta} - e^{-(k+1)\delta}} e^{-k\delta} \tag{6}$$

WET SEASON

Within the wet season, there are 2 different periods:

1) **First part of the wet season (during *b* days):** Period in which the probability of death AND the probability of introduction are occurring simultaneously.
2) **Second part of the wet season (during *s - b* days):** Period in which only the probability of death occurs (same as dry season).

Second part of the wet season (during *s - b* days)

Only death occurs during this period. Therefore, similar to the dry season, the population size on day *n* is:

$$P_n = P_b e^{-n\delta} \tag{7}$$

And the population size at the end of the wet season is:

$$P_{W'} = P_b e^{-(s-b)\delta} \tag{8}$$

The population at the last day of the wet season, $P_{W'}$, must equal to P_D:

$$P_{W'} = P_D$$

Using equations (4) and (8),

$$P_b e^{-(s-b)\delta} = \frac{kA \times Mean_{D-dry}\left(1 - e^{-\delta}\right)}{e^{-\delta} - e^{-(k+1)\delta}}$$

$$P_b = \frac{kA \times Mean_{D-dry}\left(1 - e^{-\delta}\right)}{e^{-\delta} - e^{-(k+1)\delta}} e^{(s-b)\delta}$$

With $k = N - s$

$$P_b = \frac{(N-s)A \times Mean_{D\_dry}\left(1 - e^{-\delta}\right)}{e^{-\delta} - e^{-(N-s+1)\delta}} e^{(s-b)\delta} \tag{9}$$

Using equation (7), the sum of daily densities during the second part of the wet season:

$$Sum_{D\_wet2} = \frac{\sum_{n=1}^{s-b} P_n}{A}$$

$$Sum_{D\_wet2} = \frac{P_b \sum_{n=1}^{s-b} e^{-n\delta}}{A}$$

Applying Equation (3) for the geometric series yields

$$Sum_{D\_wet2} = \frac{P_b}{A} \frac{e^{-\delta} - e^{-(s-b+1)\delta}}{1 - e^{-\delta}} \tag{10}$$

First part of the wet season (during *b* days)

On day 1, the population size is:

$$P_1 = P_W - P_W\left(1 - e^{-\delta}\right) + CP_b$$
$$P_1 = P_W e^{-\delta} + CP_b$$

Here, the first term on the right hand sider is the initial population of the dingoes subtracting the dingoes that died on day 1. The second term, CP_b, is the number of dingoes born in day 1 of the wet season.
On day 2, the population size is:

$$P_2 = P_1 e^{-\delta} + CP_b$$
$$P_2 = \left(P_W e^{-\delta} + CP_b\right)e^{-\delta} + CP_b$$
$$P_2 = P_W e^{-2\delta} + CP_b\left(1 + e^{-\delta}\right)$$

By recursion, on day *n*, the population size is:

$$P_n = P_W e^{-n\delta} + CP_b \sum_{m=0}^{n-1} e^{-m\delta}$$

The geometric series with index starting at zero converges according to:

$$\sum_{k=0}^{n} r^k = \frac{1 - r^{n+1}}{1 - r}$$

Thus,

$$P_n = P_W e^{-n\delta} + CP_b \frac{1 - e^{-n\delta}}{1 - e^{-\delta}} \qquad (11)$$

Since $P_W = P_{D'}$ and using equation (2) to replace $P_{D'}$:

$$P_W = P_D e^{-k\delta}$$

Since $P_{W'} = P_D$, and using equation (8) to replace P_D in the above equation:

$$P_W = \left\{P_b e^{-(s-b)\delta}\right\} e^{-k\delta}$$
$$P_W = P_b e^{-(s-b+k)\delta}$$

With this result, equation (11) takes the form

$$P_n = P_b e^{-(s-b+k+n)\delta} + CP_b \frac{1 - e^{-n\delta}}{1 - e^{-\delta}}$$

$$P_n = P_b \left\{ e^{-(N-b+n)\delta} + C\frac{1 - e^{-n\delta}}{1 - e^{-\delta}} \right\}$$

At day *b*, the population size is:

$$P_b = P_b \left\{ e^{-N\delta} + C\frac{1 - e^{-b\delta}}{1 - e^{-\delta}} \right\}$$
$$1 = e^{-N\delta} + C\frac{1 - e^{-b\delta}}{1 - e^{-\delta}}$$

The result provides the solution for term C in this formula.

$$1 = e^{-N\delta} + C\frac{1 - e^{-b\delta}}{1 - e^{-\delta}}$$

$$C = \frac{\left(1 - e^{-N\delta}\right)\left(1 - e^{-\delta}\right)}{1 - e^{-b\delta}} \qquad (12)$$

The sum of daily densities during the first part of the wet season (from day 1 to day b) becomes:

$$Sum_{D\_wet1} = \frac{\sum_{n=1}^{b} P_n}{A} = \frac{P_b}{A}\sum_{n=1}^{b}\left\{e^{-(N-b+n)\delta} + C\frac{1 - e^{-n\delta}}{1 - e^{-\delta}}\right\}$$

$$Sum_{D\_wet1} = \frac{P_b}{A}e^{-(N-b)\delta}\sum_{n=1}^{b} e^{-n\delta} + \frac{CP_b}{A(1 - e^{-\delta})}\sum_{n=1}^{b}\left(1 - e^{-n\delta}\right)$$

Using equation (3) for the geometric series, and equation (12) for C, yields

$$Sum_{D\_wet1} = \frac{P_b}{A}e^{-(N-b)\delta}\frac{e^{-\lambda} - e^{-(b+1)\delta}}{1 - e^{-\delta}} + \frac{P_b}{A}\frac{1}{1 - e^{-\delta}}\frac{\left(1 - e^{-N\delta}\right)\left(1 - e^{-\delta}\right)}{1 - e^{-b\delta}}\left(b - \frac{e^{-\delta} - e^{-(b+1)\delta}}{1 - e^{-\delta}}\right)$$

$$Sum_{D\_wet1} = \frac{P_b}{A}e^{-(N-b)\delta}\frac{e^{-\delta} - e^{-(b+1)\delta}}{1 - e^{-\delta}} + \frac{P_b}{A}\frac{\left(1 - e^{-N\delta}\right)}{1 - e^{-b\delta}}\left(b - \frac{e^{-\delta} - e^{-(b+1)\delta}}{1 - e^{-\delta}}\right) \qquad (13)$$

The average daily density during the wet season must equal:

$$Mean_{Dwet} = \frac{Sum_{D\_wet1} + Sum_{D\_wet2}}{s}$$

Substituting the expressions for the sum densities, equations (10) and (13) yields

$$Mean_{D\_wet} = \frac{P_b}{As}\left\{\frac{e^{-\delta} - e^{-(s-b+1)\delta}}{1 - e^{-\delta}} + e^{-(N-b)\delta}\frac{e^{-\delta} - e^{-(b+1)\delta}}{1 - e^{-\delta}} + \frac{\left(1 - e^{-N\delta}\right)}{1 - e^{-b\delta}}\left(b - \frac{e^{-\delta} - e^{-(b+1)\delta}}{1 - e^{-\delta}}\right)\right\}$$

The $Mean_{D\_wet}$ may be expressed in terms of $Mean_{D\_dry}$ by replacing the term P_b by equation (9).

$$Mean_{D\_wet} = \frac{1}{As}\frac{(N - s)A \times Mean_{D\_dry}\left(1 - e^{-\delta}\right)}{e^{-\delta} - e^{-(N-s+1)\delta}}e^{(s-b)\delta}\left\{\frac{e^{-\delta} - e^{-(s-b+1)\delta}}{1 - e^{-\delta}}\right.$$
$$\left. + e^{-(N-b)\delta}\frac{e^{-\delta} - e^{-(b+1)\delta}}{1 - e^{-\delta}} + \frac{\left(1 - e^{-N\delta}\right)}{1 - e^{-b\delta}}\left(b - \frac{e^{-\delta} - e^{-(b+1)\delta}}{1 - e^{-\delta}}\right)\right\}$$

$$Mean_{D\_wet} = \frac{(N-s)Mean_{D\_dry}}{s} \frac{e^{(s-b)\delta}}{e^{-\delta} - e^{-(N-s+1)\delta}} \left\{ e^{-\delta} - e^{-(s-b+1)\delta} + e^{-(N-b)\delta}\left(e^{-\lambda} - e^{-(b+1)\delta}\right) + \frac{\left(1-e^{-N\delta}\right)}{1-e^{-b\delta}}\left[b\left(1-e^{-\delta}\right) - \left(e^{-\delta} - e^{-(b+1)\delta}\right)\right] \right\}$$

$$Mean_{Dwet} = \frac{(N-s)Mean_{D\_dry}}{s} \frac{e^{(s-b)\delta}}{e^{-\delta} - e^{-(N-s+1)\delta}} \left\{ e^{-\delta} - e^{-(s-b+1)\delta} + e^{-(N-b)\delta}\left(e^{-\lambda} - e^{-(b+1)\delta}\right) + \frac{b\left(1-e^{-N\delta}\right)\left(1-e^{-\delta}\right)}{1-e^{-b\delta}} - \frac{\left(1-e^{-N\delta}\right)\left(e^{-\delta} - e^{-(b+1)\delta}\right)}{1-e^{-b\delta}} \right\} \quad (14)$$

For each simulation, the model randomly selects a value of the mean density during the dry season. This value is then multiplied by a factor of 1.0888889 to calculate the mean density during the wet season, as this corresponds to the ratio found between both mean density results from the camera-trap study. Using equation (14), with the selected and calculated values of mean densities during the dry and wet seasons, $N = 365$ days, s =180 days and b = 90 days, the model numerically calculates the value for the death rate, δ, in order to satisfy all above conditions and ensure that the population size remains stable from one year to another.

Home range distribution

For each dingo, based on its own selected 95% bivariate home range size estimate, a circular bivariate (2 dimensional) normal distribution will be fitted in view of obtaining a probability density function (referred to as home range distribution, in the manuscript). The probability density function for dingo i, $P_i(x,y)$, can be described by the following equation:

$$P_i(x,y) = \frac{1}{2\pi\sigma_{xi}\sigma_{yi}\sqrt{1-\rho_i^2}} \exp\left\{-\frac{1}{2(1-\rho_i^2)}\left[\frac{(x-\mu_{xi})^2}{\sigma_{xi}^2} + \frac{(y-\mu_{yi})^2}{\sigma_{yi}^2} - \frac{2\rho_i(x-\mu_{xi})(y-\mu_{yi})}{\sigma_{xi}\sigma_{yi}}\right]\right\}$$

We will consider the function to be centered at the origin $\mu_{xi} = \mu_{yi} = 0$. Since we want to describe a circular function, the correlation $\rho_i = 0$ and the standard deviations $\sigma_{xi} = \sigma_{yi} = \sigma_i$. With these conditions, the probability density distribution simplifies to:

$$P_i(x,y) = \frac{1}{2\pi\sigma_i^2} \exp\left\{-\frac{x^2+y^2}{2\sigma_i^2}\right\}$$

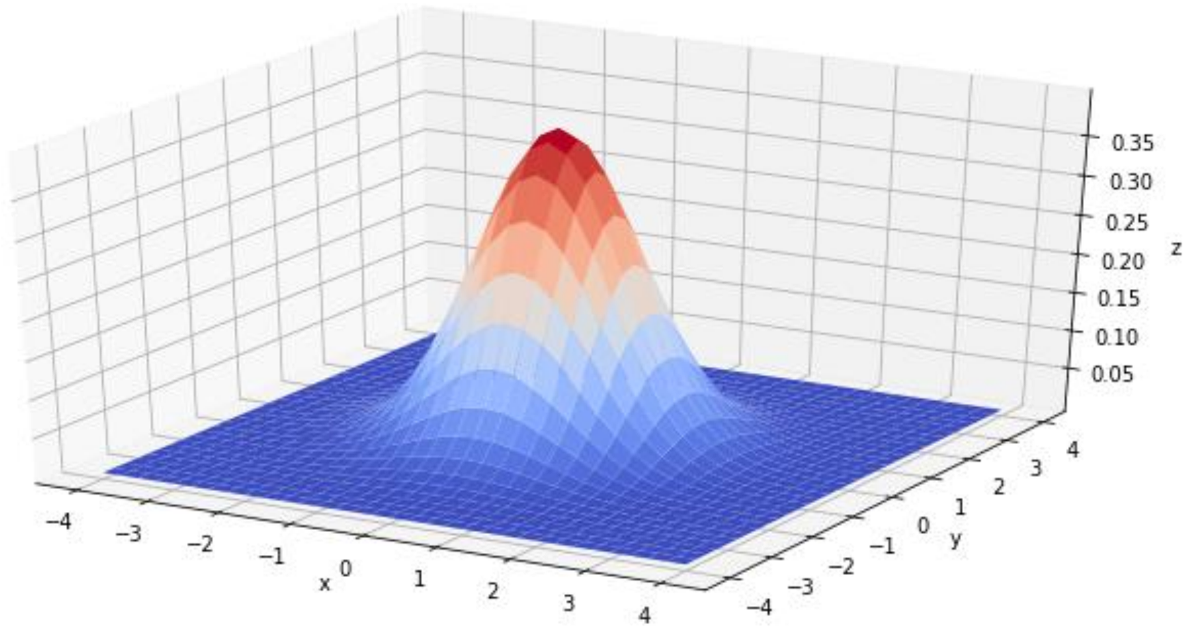

Figure B. Example of the Probability density function of a dingo, centered at the centroid point of its home range (0,0)

The integral of $P_i(x,y)$ over the entire plane gives a probability of 1.

$$P_i(x,y) = \frac{1}{2\pi\sigma_i^2} \int_{-\infty}^{\infty} dx \int_{-\infty}^{\infty} dy \exp\left\{-\frac{x^2+y^2}{2\sigma_i^2}\right\} = 1$$

In polar coordinates, (r, θ) the integral takes the form

$$P_i(r,\theta) = \frac{1}{2\pi\sigma_i^2} \int_0^{2\pi} d\theta \int_0^{\infty} dr r \exp\left\{-\frac{r^2}{2\sigma_i^2}\right\}$$

The value of σ will be calculated by assuming that the probability of finding the dingo inside the home range estimate is 95%. Therefore, the probability of finding dingo *i* within a circle of radius R_{95}, corresponding to the area of the 95% home range estimate is

$$P_i(r,\theta) = \frac{1}{2\pi\sigma_i^2} \int_0^{2\pi} d\theta \int_0^{R_{95}} dr r \exp\left\{-\frac{r^2}{2\sigma_i^2}\right\} = 0.95$$

Integrating and simplifying yields,

$$\exp\left\{-\frac{{R_{95}}^2}{2\sigma_i^2}\right\} = 0.05$$

$$\sigma_i^2 = -\frac{{R_{95}}^2}{2\ln(0.05)}$$

$$\sigma_i = 0.4085 R_{95} \tag{15}$$

The standard error will be calculated for each dingo, based on its selected seasonal 95% home range value.

Probability of relocation in a new vacant home range area

We assume that dingo i will relocate to a new home range centroid if it found itself within an arc-shaped area of dimensions *u* around the location of the new home range centroid, and if the distance *d* between the dingo's home range centroid and the new location is larger than R_{95}. The probability of dingo i relocating is therefore calculated as the integral of the probability density function over the area formed by this arc, as followed:

$$PR_i = \frac{1}{2\pi\sigma_i^2}\int_{-Arctan(u/d)}^{Arctan(u/d)} d\theta \int_{d-u}^{d+u} r\, exp\left(-\frac{r^2}{2\sigma_i^2}\right) dr$$

Here d equals the distance between the dingo *i* centroid and the new vacant centroid and σ_i equals the standard deviation of the home range distribution for dingo *i*.

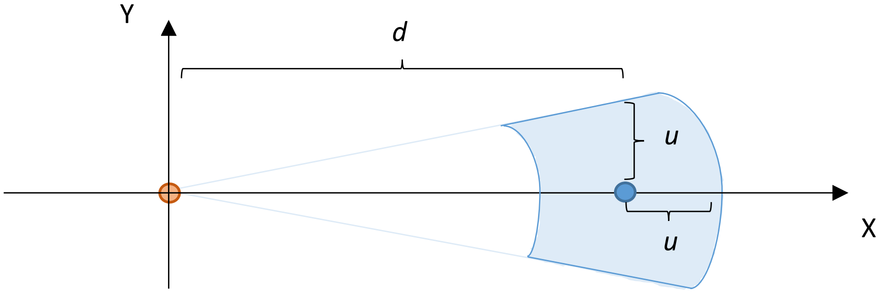

Figure C. Two dimensional view (XY) of the arc-shaped area (blue zone) of integration to calculate the probability of relocation. The dingo's home range centroid and the new vacant centroid are represented by the red and blue points, respectively, and separated by a distance *d*. The probability density function of the dingo will be integrated over the arc-shaped area, which is of dimension *u* (In the model, *u* takes a value of 1km).

Integrating yields

$$PR_\mathrm{i} = \frac{1}{\pi}\mathrm{Arctan}\left(\frac{\mathrm{u}}{d}\right)\left\{\exp\left(-\frac{(d-\mathrm{u})^2}{2\sigma_i^2}\right) - \exp\left(-\frac{(d+\mathrm{u})^2}{2\sigma_i^2}\right)\right\}$$

The probability of dingo *i* relocating (PR_i) is based on the probability density function of the dingo. This function describes the probability of a dingo occurring at one location within a time frame which corresponds to the number of days, n_i, needed for the dingo to explore most of its home range area (and therefore the time needed to build its probability density function). We will assume that relocation is an independent event from one day to another. Consequently, the daily probability of relocation therefore becomes:

$$PR_{\mathrm{i}\_daily} = 1 - \sqrt[n_i]{1 - PR_\mathrm{i}}$$

$$PR_{i\_daily} = 1 - \sqrt[n_i]{1 - \frac{1}{\pi}Arctan\left(\frac{u}{d}\right)\left(exp\left(-\frac{(d-u)^2}{2\sigma_i^2}\right) - exp\left(-\frac{(d+u)^2}{2\sigma_i^2}\right)\right)} \quad (16)$$

N.B. As the distance *d* increases to infinity, the arc-shaped area will slowly take the shape of a square of $4u^2$ dimensions.

Probability of contact

In order to compute the probability of two dingoes from different packs meeting, we need to define a "position function", which is equal to the square root of the probability density function (dimension = 1/distance). The position function of dingo i centered at position $(0,0)$ is:

$$p_i(x,y) = \frac{1}{\sqrt{2\pi}\sigma_i}\exp\left\{-\frac{x^2+y^2}{4\sigma_i^2}\right\}$$

The probability of contact between dingo i and dingo j, which have centroid points separated by a distance d, will be calculated by integrating the product of the position function of dingo i (centered at $\mu_{xi} = 0$ and $\mu_{yi} = 0$) and the position function of dingo j (centered at $\mu_{xj} = d$ and $\mu_{yj} = 0$), as follows:

$$p_i(x,y)p_j(x,y) = \frac{1}{\sqrt{2\pi}\sigma_i}\exp\left\{-\frac{x^2+y^2}{4\sigma_i^2}\right\} \times \frac{1}{\sqrt{2\pi}\sigma_j}\exp\left\{-\frac{(x-d)^2+y^2}{4\sigma_j^2}\right\}$$

$$PC_{ij} = \frac{1}{2\pi\sigma_i\sigma_j}\int_{-\infty}^{\infty} dx\exp\left\{-\frac{x^2}{4\sigma_i^2}-\frac{(x-d)^2}{4\sigma_j^2}\right\}\int_{-\infty}^{\infty} dy\exp\left\{-\left(\frac{1}{\sigma_i^2}+\frac{1}{\sigma_j^2}\right)\frac{y^2}{4}\right\}$$

$$PC_{ij} = \frac{1}{2\pi\sigma_i\sigma_j}\int_{-\infty}^{\infty} dx\exp\left\{-\frac{x^2}{4\sigma_i^2}-\frac{x^2-2xd+d^2}{4\sigma_j^2}\right\}\int_{-\infty}^{\infty} dy\exp\left\{-\left(\frac{\sigma_i^2+\sigma_j^2}{\sigma_i^2\sigma_j^2}\right)\frac{y^2}{4}\right\}$$

$$PC_{ij} = \frac{1}{2\pi\sigma_i\sigma_j}\int_{-\infty}^{\infty} dx\exp\left\{-\left(\frac{\sigma_i^2+\sigma_j^2}{\sigma_i^2\sigma_j^2}\right)\frac{x^2}{4}+\frac{2xd}{4\sigma_j^2}-\frac{d^2}{4\sigma_j^2}\right\}\int_{-\infty}^{\infty} dy\exp\left\{-\left(\frac{\sigma_i^2+\sigma_j^2}{\sigma_i^2\sigma_j^2}\right)\frac{y^2}{4}\right\}$$

$$PC_{ij} = \frac{1}{2\pi\sigma_i\sigma_j}\exp\left\{-\frac{d^2}{4\sigma_j^2}\right\}\int_{-\infty}^{\infty} dx\exp\left\{-\frac{1}{4}\left(\frac{\sigma_i^2+\sigma_j^2}{\sigma_i^2\sigma_j^2}\right)x^2+\frac{d}{2\sigma_j^2}x\right\}\int_{-\infty}^{\infty} dy\exp\left\{-\left(\frac{\sigma_i^2+\sigma_j^2}{\sigma_i^2\sigma_j^2}\right)\frac{y^2}{4}\right\}$$

The solutions integrals are given by the formulae:

(i) $\int_{-\infty}^{\infty} dx e^{-ax^2} = \sqrt{\frac{\pi}{a}}$

(ii) $\int_{-\infty}^{\infty} dx e^{-ax^2+bx} = \sqrt{\frac{\pi}{a}}e^{\frac{b^2}{4a}}$

$$PC_{ij} = \frac{1}{2\pi\sigma_i\sigma_j}\exp\left\{-\frac{d^2}{4\sigma_j^2}\right\}\left[\sqrt{\frac{4\pi\sigma_i^2\sigma_j^2}{\sigma_i^2+\sigma_j^2}}\exp\left\{\frac{d^2}{4\sigma_j^4}\cdot\frac{\sigma_i^2\sigma_j^2}{\sigma_i^2+\sigma_j^2}\right\}\right]\sqrt{\frac{4\pi\sigma_i^2\sigma_j^2}{\sigma_i^2+\sigma_j^2}}$$

$$PC_{ij} = \frac{1}{2\pi\sigma_i\sigma_j} \exp\left\{-\frac{d^2}{4\sigma_j^2}\right\} \frac{4\pi\sigma_i^2\sigma_j^2}{\sigma_i^2 + \sigma_j^2} \exp\left\{\frac{d^2}{4\sigma_j^2} \cdot \frac{\sigma_i^2}{\sigma_i^2 + \sigma_j^2}\right\}$$

$$PC_{ij} = \frac{2\sigma_i\sigma_j}{\sigma_i^2 + \sigma_j^2} \exp\left\{\frac{d^2}{4\sigma_j^2}\left(\frac{\sigma_i^2}{\sigma_i^2 + \sigma_j^2} - 1\right)\right\}$$

$$PC_{ij} = \frac{2\sigma_i\sigma_j}{\sigma_i^2 + \sigma_j^2} \exp\left\{\frac{d^2}{4\sigma_j^2}\left(-\frac{\sigma_j^2}{\sigma_i^2 + \sigma_j^2}\right)\right\}$$

$$PC_{ij} = \frac{2\sigma_i\sigma_j}{\sigma_i^2 + \sigma_j^2} \exp\left\{-\frac{d^2}{4\left(\sigma_i^2 + \sigma_j^2\right)}\right\}$$

From this equation, we can see that the probability of contact decreases as the distance between centroids increases ($PC_{ij} \rightarrow 0$ as $d \rightarrow \infty$).

Similar to the probability of relocation, PC_{AB} describes the probability of contact between two dingoes during a time period, n_{ij}, equal to the average time needed for both dingoes to explore most of their home range area. The daily probability of contact between dingo A and dingo B is therefore equal to:

$$PC_{ij\_daily} = 1 - \sqrt[n_{ij}]{1 - PC_{ij}}$$

$$PC_{ij\_daily} = 1 - \sqrt[n_{ij}]{1 - \frac{2\sigma_i\sigma_j}{\sigma_i^2 + \sigma_j^2} \exp\left\{-\frac{d^2}{4\left(\sigma_i^2 + \sigma_j^2\right)}\right\}} \quad (17)$$

With $n_{ij} = \frac{n_i + n_j}{\mathbf{2}}$

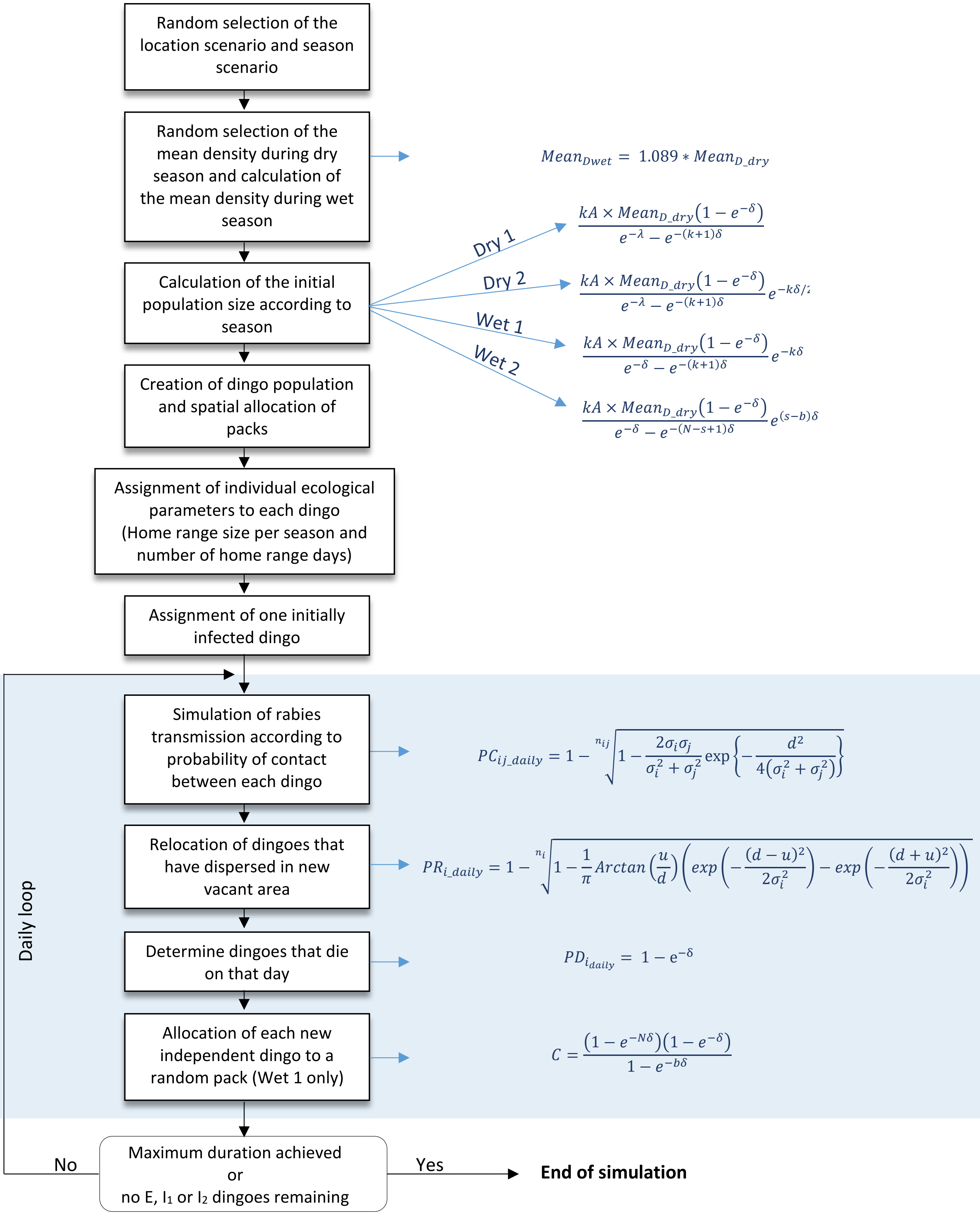

Figure D. Flow diagram illustrating the main steps, with the corresponding equations, for one simulation in the model. The steps found within the shaded area in blue are repeated at each daily time-step during the simulation.

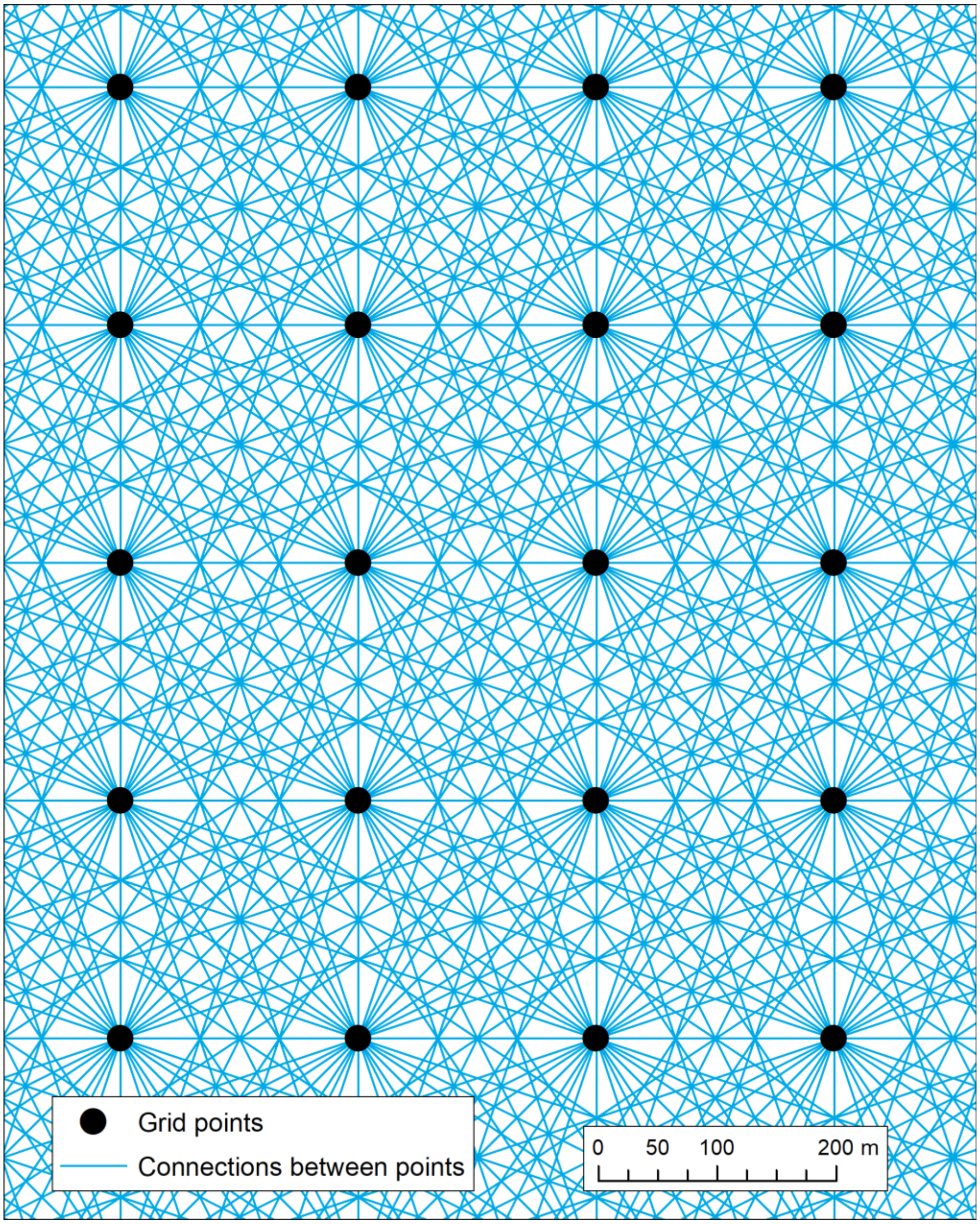

Figure E. Representation of a very small subset of grid points and the network resulting from the connections between each point and its 32 nearest neighboring points in all directions. The connections represent the edges in which dingoes were allowed to travel from one point to another within the network.
