# Supplementary material for: Could a rabies incursion spread in the northern Australian dingo population? Development of a spatial stochastic simulation model
Source: PLoS Negl Trop Dis. 2021 Feb 12;15(2):e0009124. doi: 10.1371/journal.pntd.0009124 (PMC7906478; doi:10.1371/journal.pntd.0009124)
Supplement: S1 Table — (PDF) [file pntd.0009124.s002.pdf]

Table S1. Outcome summary statistics (median, 2.5th percentile and 97.5th percentile) for each location and season scenario, when considering the subset of simulations which resulted in more than one pack infected and when considering all simulations from a spatial rabies spread model within a dingo population in the Northern Peninsula Area of Queensland, Australia.

**Outcome measure**	**Simulations with more than 1 pack infected**				**All simulations**			
	**Median**	**2.5th percentile**	**97.5th percentile**	**Number simulations**	**Median**	**2.5th percentile**	**97.5th percentile**	**Number simulations**
Number of infected dingoes								
Community	17	2	108	36332	4	1	98	66687
Hunter	25	2	71	37920	5	1	60	66483
Random	26	2	104	40951	7	1	95	66830
Dry1	30	2	110	33482	12	1	103	50192
Dry2	25	2	97	34127	11	1	90	50274
Wet1	16	2	82	29180	5	1	70	49730
Wet2	13	2	102	18414	2	1	82	49804
All scenarios	22	2	101	115203	5	1	91	200000
Proportion of packs infected								
Community	17.4	4	68.1	36332	4.3	2.0	62.2	66687
Hunter	11.4	4	44.4	37920	4.3	2.0	37.8	66483
Random	17.4	4	65.4	40951	6.1	2.0	60.8	66830
Dry1	20.8	4.1	69.8	33482	8.9	2.0	66.0	50192
Dry2	17.4	4	59.6	34127	8.9	2.0	55.8	50274
Wet1	10.6	3.9	50.0	29180	4.3	1.9	43.2	49730
Wet2	8.7	4	65.1	18414	2.3	2.0	52.3	49804
All scenarios	14.8	4	63.6	115203	4.4	2.0	57.8	200000
R0 at dingo level								
Community	2	1	8	36332	1	0	7	66687
Hunter	3	1	8	37920	1	0	7	66483
Random	3	1	9	40951	1	0	8	66830

Dry1	3	1	9	33482	2	0	8	50192
Dry2	3	1	9	34127	2	0	8	50274
Wet1	3	1	8	29180	1	0	7	49730
Wet2	3	1	6	18414	1	0	5	49804
All scenarios	3	1	8	115203	1	0	7	200000
R0 at pack level								
Community	2	1	5	36332	1	0	4	66687
Hunter	2	1	6	37920	1	0	5	66483
Random	2	1	6	40951	1	0	6	66830
Dry1	2	1	6	33482	1	0	5	50192
Dry2	2	1	6	34127	1	0	6	50274
Wet1	2	1	5	29180	1	0	5	49730
Wet2	1	1	4	18414	0	0	3	49804
All scenarios	2	1	6	115203	1	0	5	200000
Area of infection								
Community	211.5	24.0	721.0	36332	66.0	2.4	661.5	66687
Hunter	144.6	23.6	475.4	37920	68.4	2.3	410.2	66483
Random	201.3	22.9	686.3	40951	82.5	2.3	637.6	66830
Dry1	252.0	33.7	741.9	33482	131.6	6.7	705.6	50192
Dry2	212.4	36.3	620.6	34127	131.0	7.7	583.3	50274
Wet1	122.4	24.0	529.3	29180	68.3	5.4	448.4	49730
Wet2	100.5	11.8	677.7	18414	21.5	2.1	562.6	49804
All scenarios	177.5	23.5	673.8	115203	71.6	2.3	613.0	200000
Duration of outbreak								
Community	210	40	523	36332	89	9	470	66687
Hunter	167	39	439	37920	92	9	388	66483
Random	205	41	508	40951	116	9	465	66830

Dry1	222	42	495	33482	140	9	458	50192
Dry2	212	42	492	34127	141	9	451	50274
Wet1	159	37	501	29180	105	9	453	49730
Wet2	176	40	506	18414	57	8	440	49804
All scenarios	191	40	498	115203	98	9	448	200000
Disease speed								
Community	0.538	0.183	1.789	36332	0.267	0	1.376	66687
Hunter	0.496	0.152	1.746	37920	0.254	0	1.406	66483
Random	0.510	0.174	1.632	40951	0.327	0	1.346	66830
Dry1	0.551	0.198	1.668	33482	0.407	0	1.417	50192
Dry2	0.551	0.180	1.824	34127	0.400	0	1.539	50274
Wet1	0.509	0.154	1.820	29180	0.284	0	1.468	49730
Wet2	0.410	0.144	1.487	18414	0.000	0	0.984	49804
All scenarios	0.516	0.167	1.716	115203	0.286	0	1.377	200000
