# Supplementary figures and images for: Could a rabies incursion spread in the northern Australian dingo population? Development of a spatial stochastic simulation model
Source: PLoS Negl Trop Dis. 2021 Feb 12;15(2):e0009124. doi: 10.1371/journal.pntd.0009124 (PMC7906478; doi:10.1371/journal.pntd.0009124)

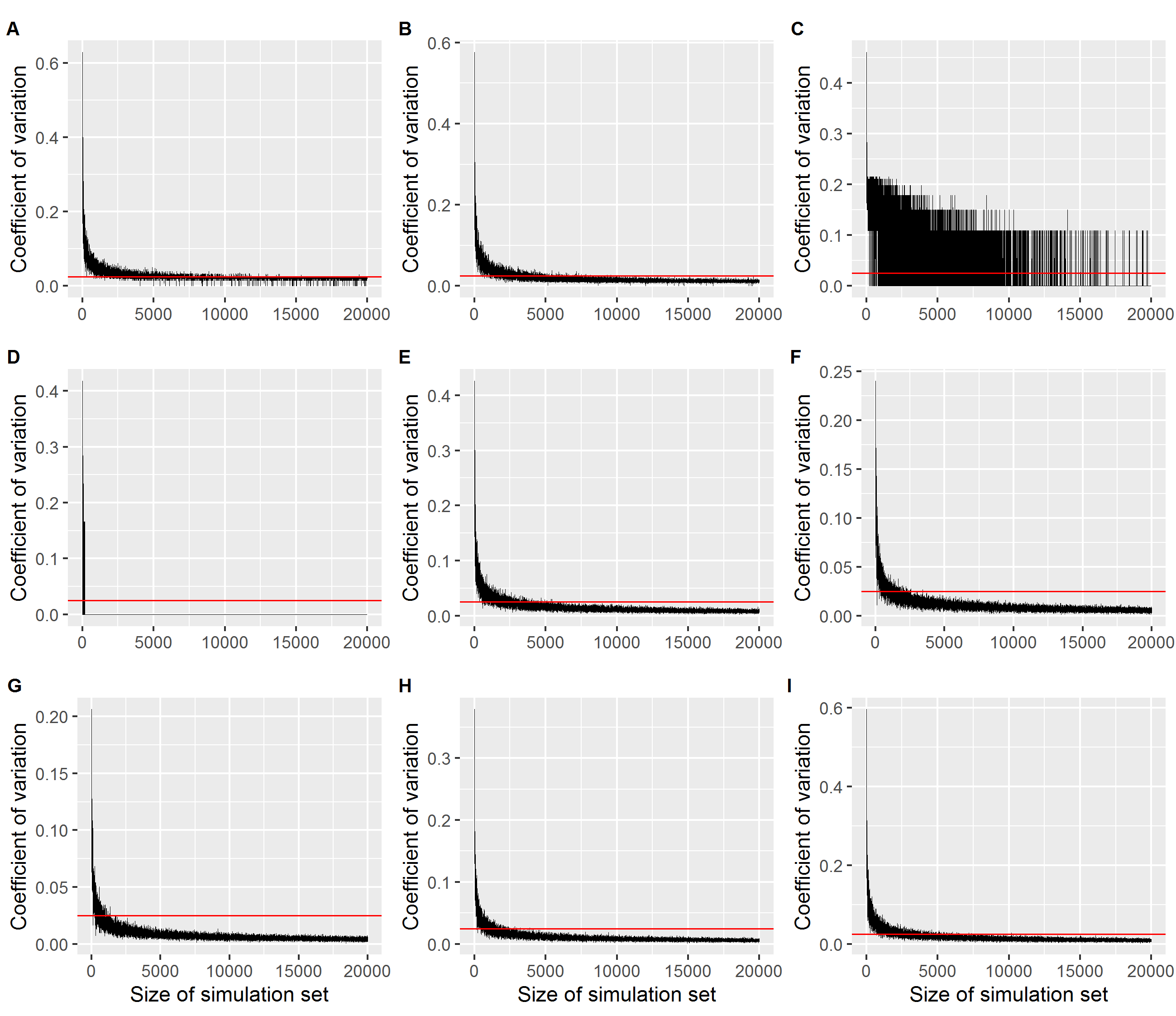

Supplement: S1 Fig — The size of simulation set represents the increasing number of simulations (x axis) in each of the 10 sets of output simulations. The outputs include (A) the number of infected dingo, (B) the proportion of infected packs, (C) R0 at the dingo level, (D) R0 at the pack level, (D) the area of infection, (E) the duration of outbreak, (F) the speed of disease spread, (G) the binary outcome indicating whether or not the infection has spread to more than one pack and (H) the binary outcome indicating whether or not the infection has spread to at least 10% of the initial population. The horizontal red line represents a coefficient of variation at 2.5%. (TIF) [file pntd.0009124.s003.tif]

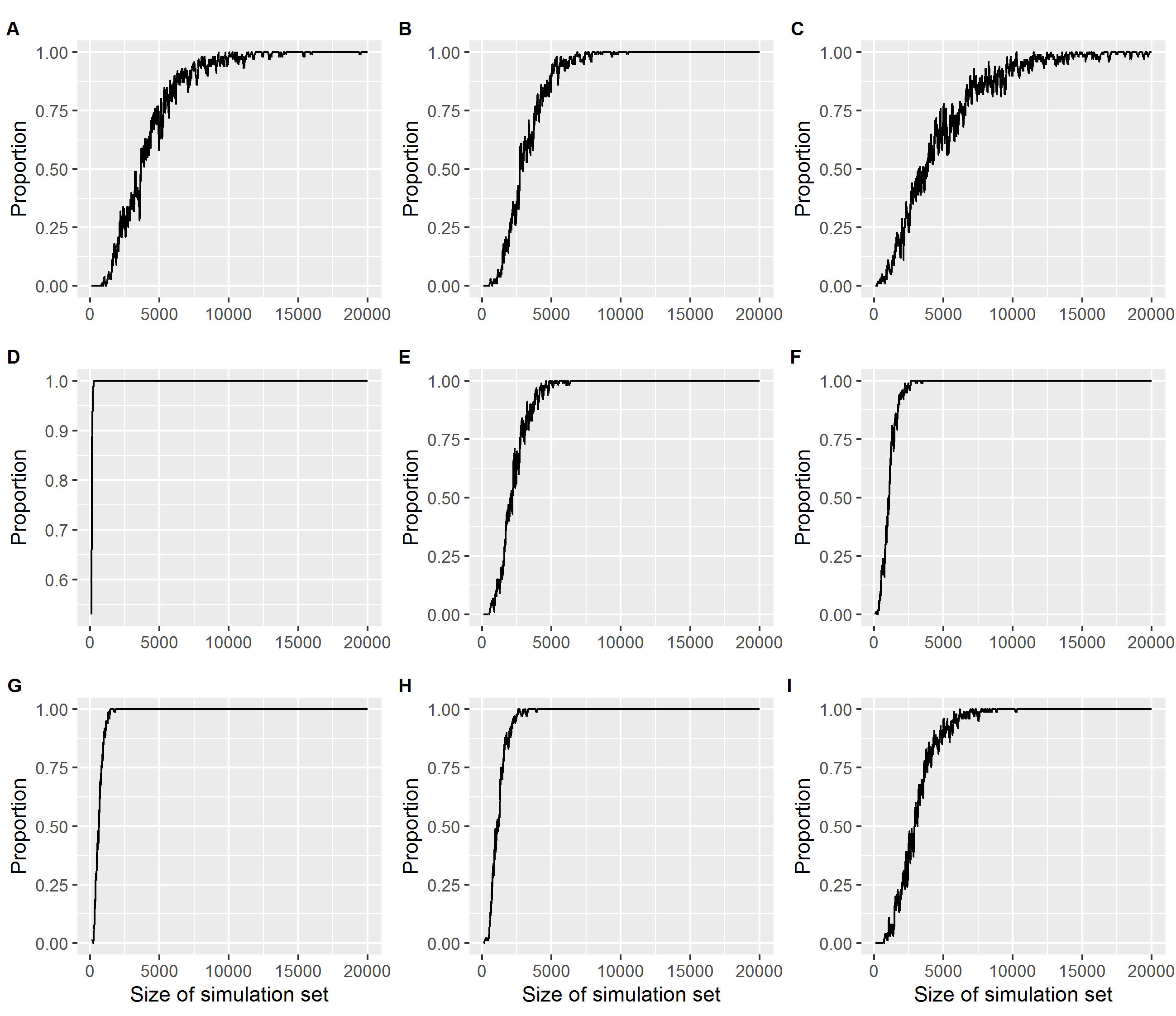

Supplement: S2 Fig — The outputs include (A) the number of infected dingo, (B) the proportion of infected packs, (C) R0 at the dingo level, (D) R0 at the pack level, (D) the area of infection, (E) the duration of outbreak, (F) the speed of disease spread, (G) the binary outcome indicating whether or not the infection has spread to more than one pack and (H) the binary outcome indicating whether or not the infection has spread to at least 10% of the initial population. (TIF) [file pntd.0009124.s004.tif]

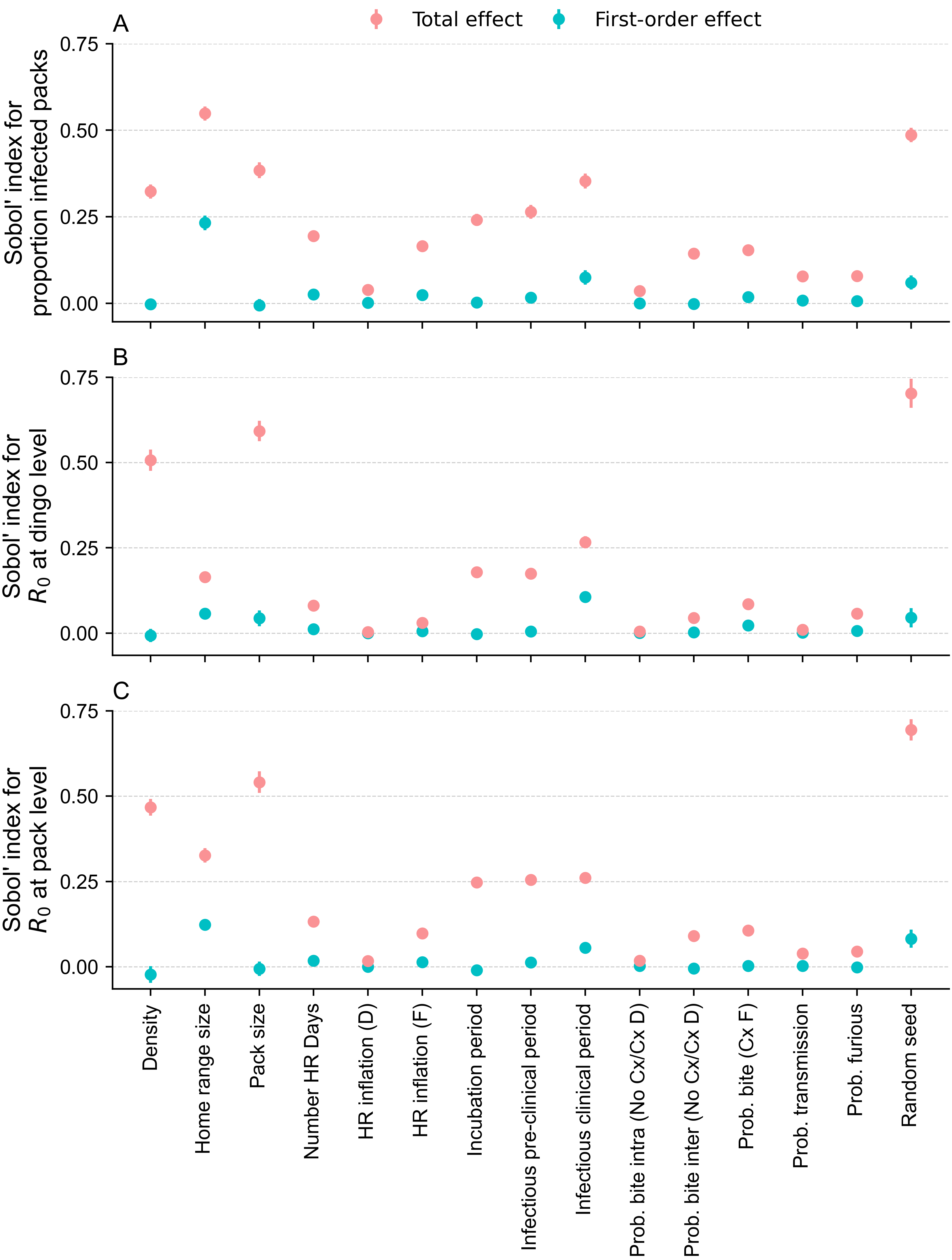

Supplement: S3 Fig — Global sensitivity indices for input parameters of a rabies spread model within a dingo population on (A) the proportion of infected packs, (B) R0 at the dingo level and (C) R0 at the pack level. The cyan and pink symbols represent the first order and total effect Sobol’ sensitivity indices. Error bars represent the 95% confidence interval for each Sobol’ index. The abbreviation “Prob.” stands for “probability”, “HR” for “Home range”, “Cx” for “clinical”, “D” for “dumb form” and “F” for “furious form”. (TIF) [file pntd.0009124.s005.tif]

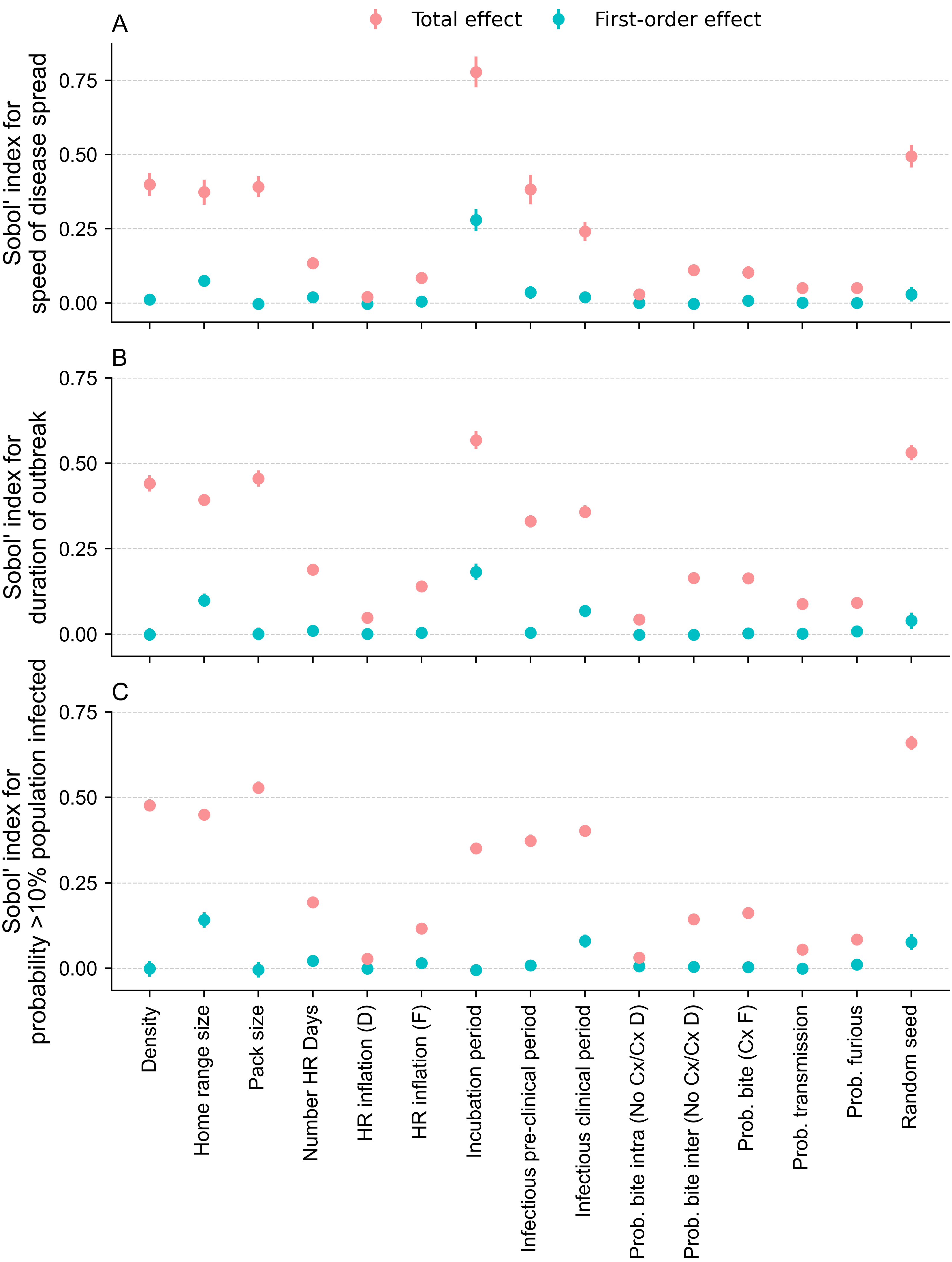

Supplement: S4 Fig — Global sensitivity indices for input parameters of a rabies spread model within a dingo population on (A) the speed of disease spread, (B) the duration of outbreak and (C) the binary outcome indicating whether or not the infection has spread to at least 10% of the initial population. The cyan and pink symbols represent the first order and total effect Sobol’ sensitivity indices. Error bars represent the 95% confidence interval for each Sobol’ index. The abbreviation “Prob.” stands for “probability”, “HR” for “Home range”, “Cx” for “clinical”, “D” for “dumb form” and “F” for “furious form”. (TIF) [file pntd.0009124.s006.tif]

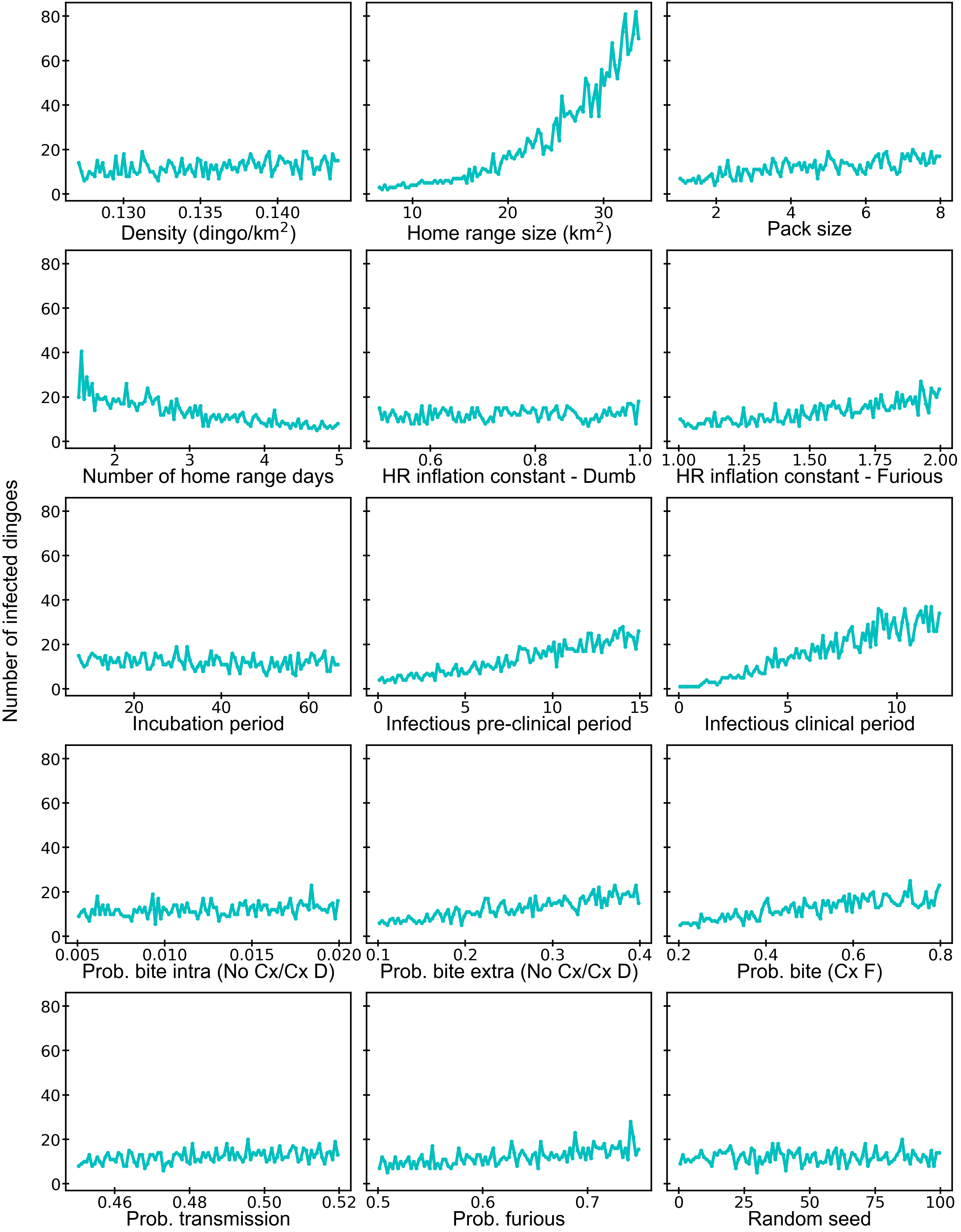

Supplement: S5 Fig — The input parameters include: Density, home range size, pack size, number of home range days, home range inflation constant for dumb infected dingoes, home range inflation constant for furious infected dingoes, incubation period, infectious pre-clinical period, infectious clinical period, probability that a non-clinical or dumb infected dingo bites another member of the pack, probability that a non-clinical or dumb infected dingo bites a dingo from another pack, probability of bite for a furious infected dingo, probability of rabies transmission given the event of a bite, probability of developing the furious form of rabies. (TIF) [file pntd.0009124.s007.tif]

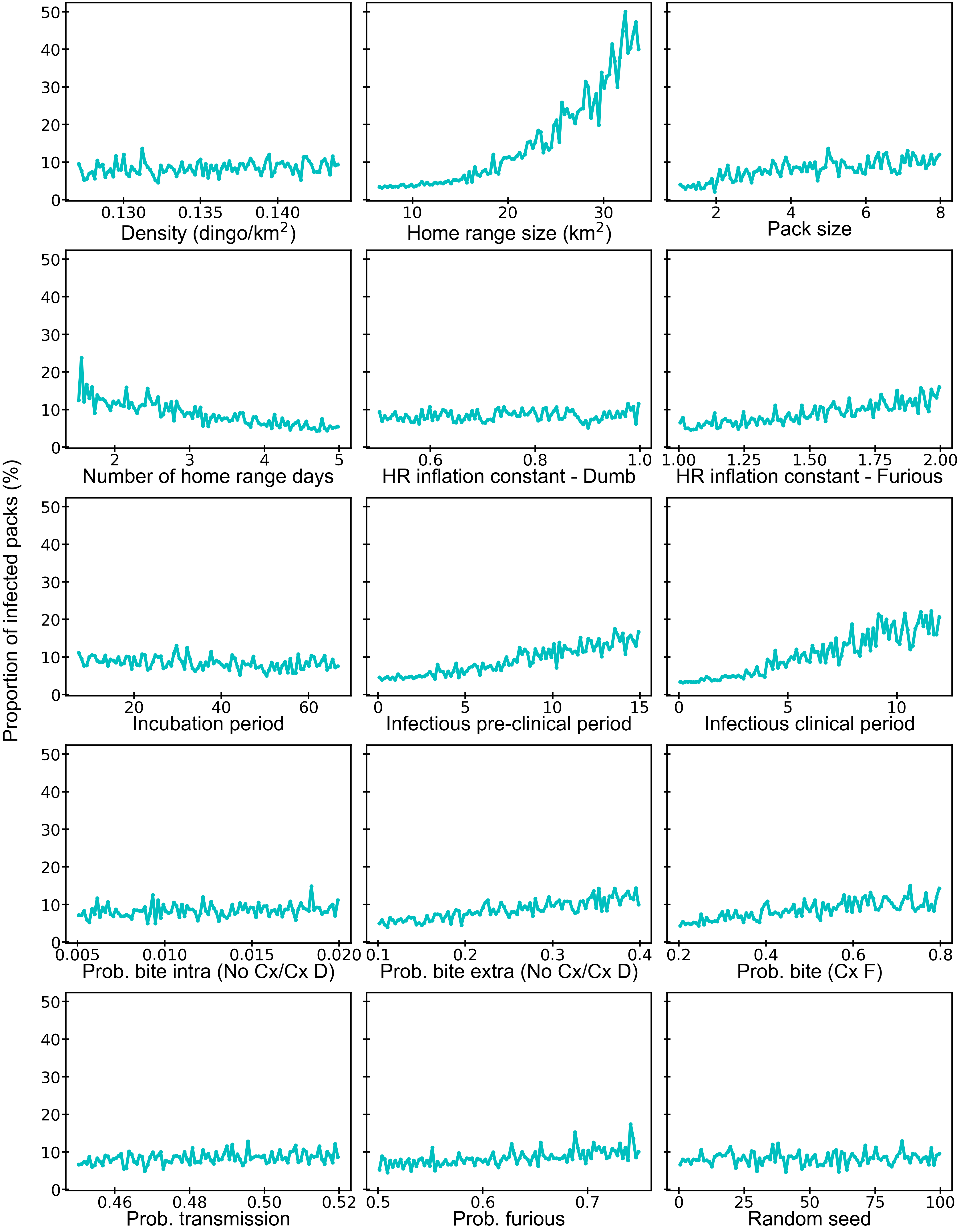

Supplement: S6 Fig — (See legend of S5 Fig). (TIF) [file pntd.0009124.s008.tif]

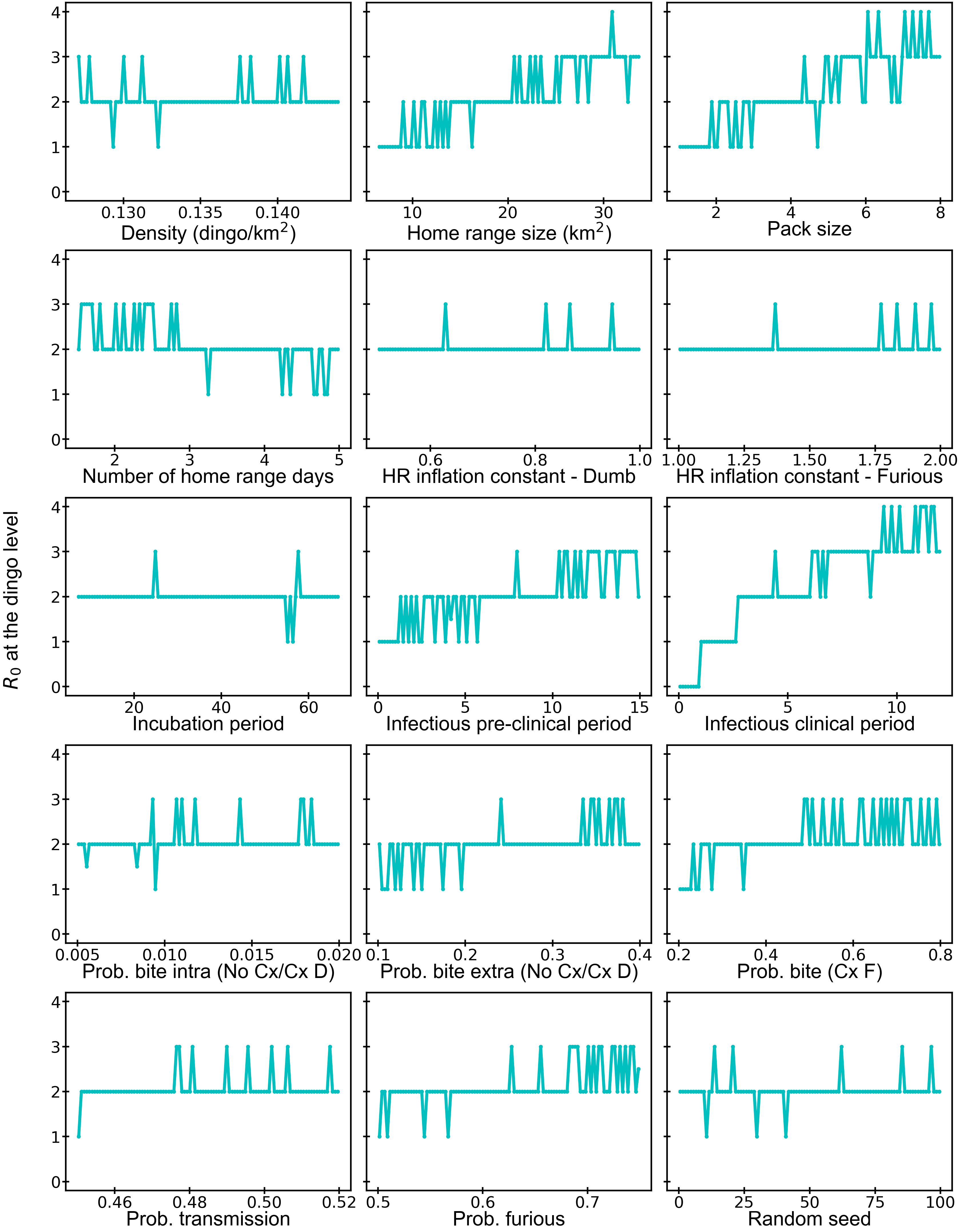

Supplement: S7 Fig — (See legend of S5 Fig). (TIF) [file pntd.0009124.s009.tif]

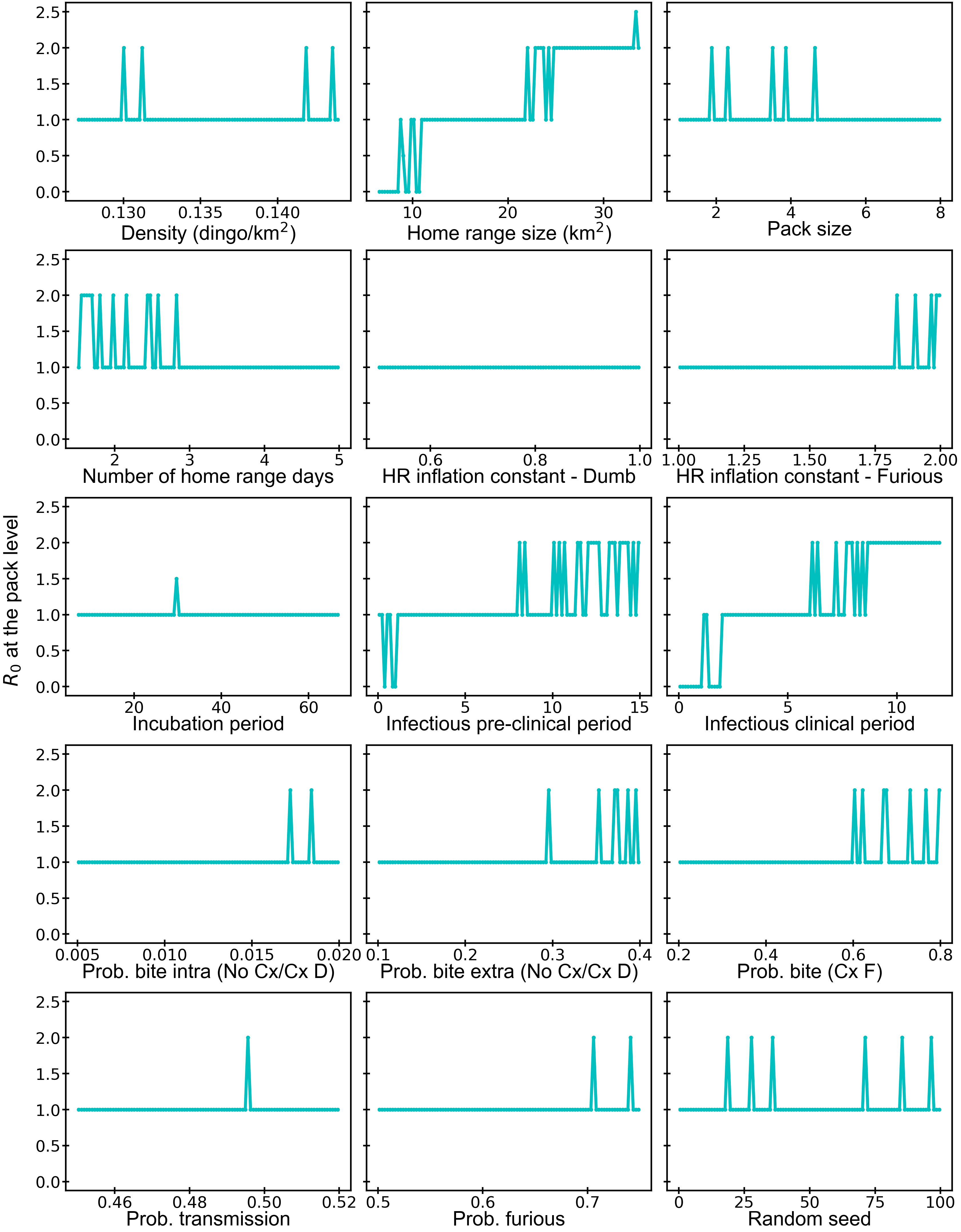

Supplement: S8 Fig — (See legend of S5 Fig) (TIF) [file pntd.0009124.s010.tif]

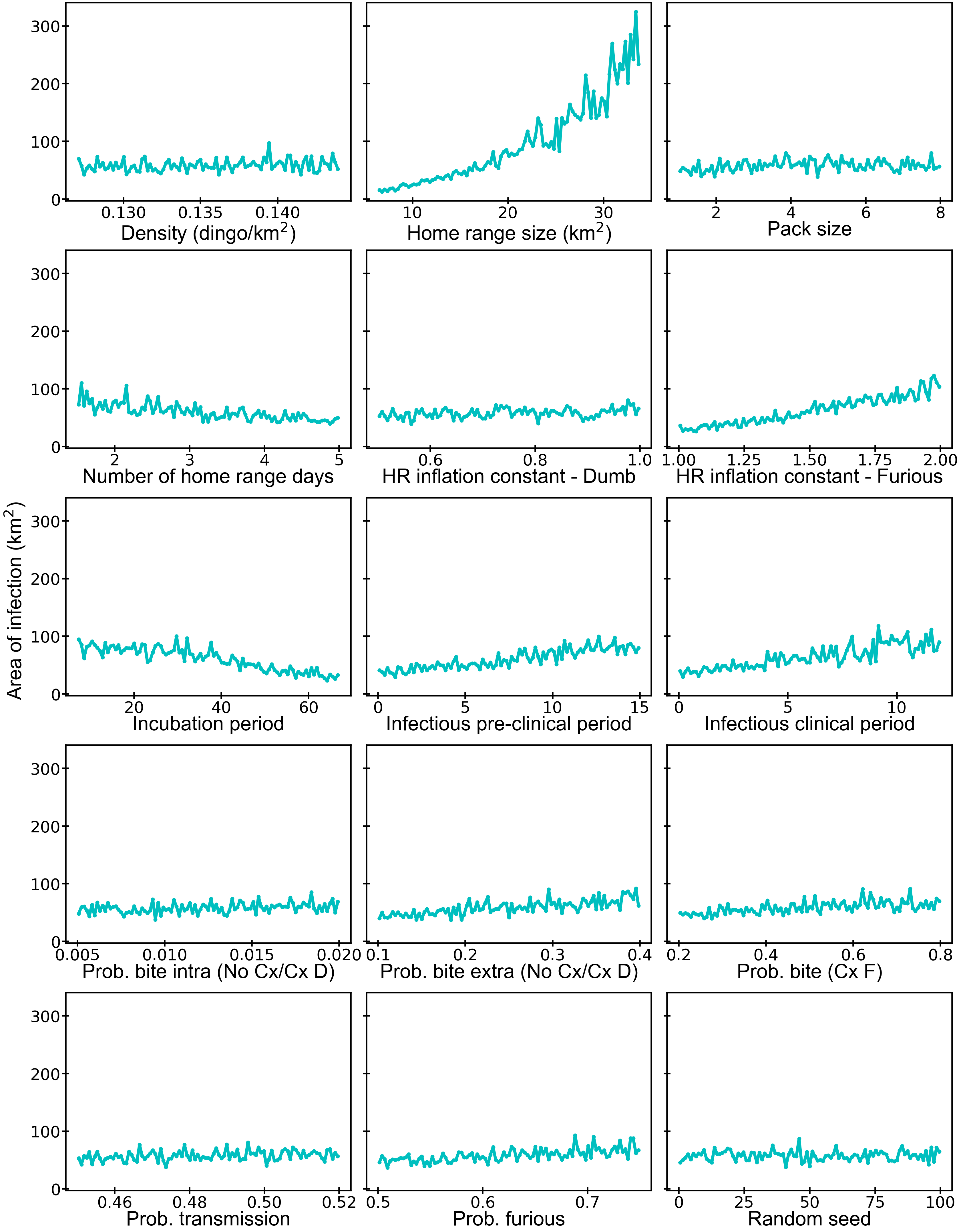

Supplement: S9 Fig — (See legend of S5 Fig). (TIF) [file pntd.0009124.s011.tif]

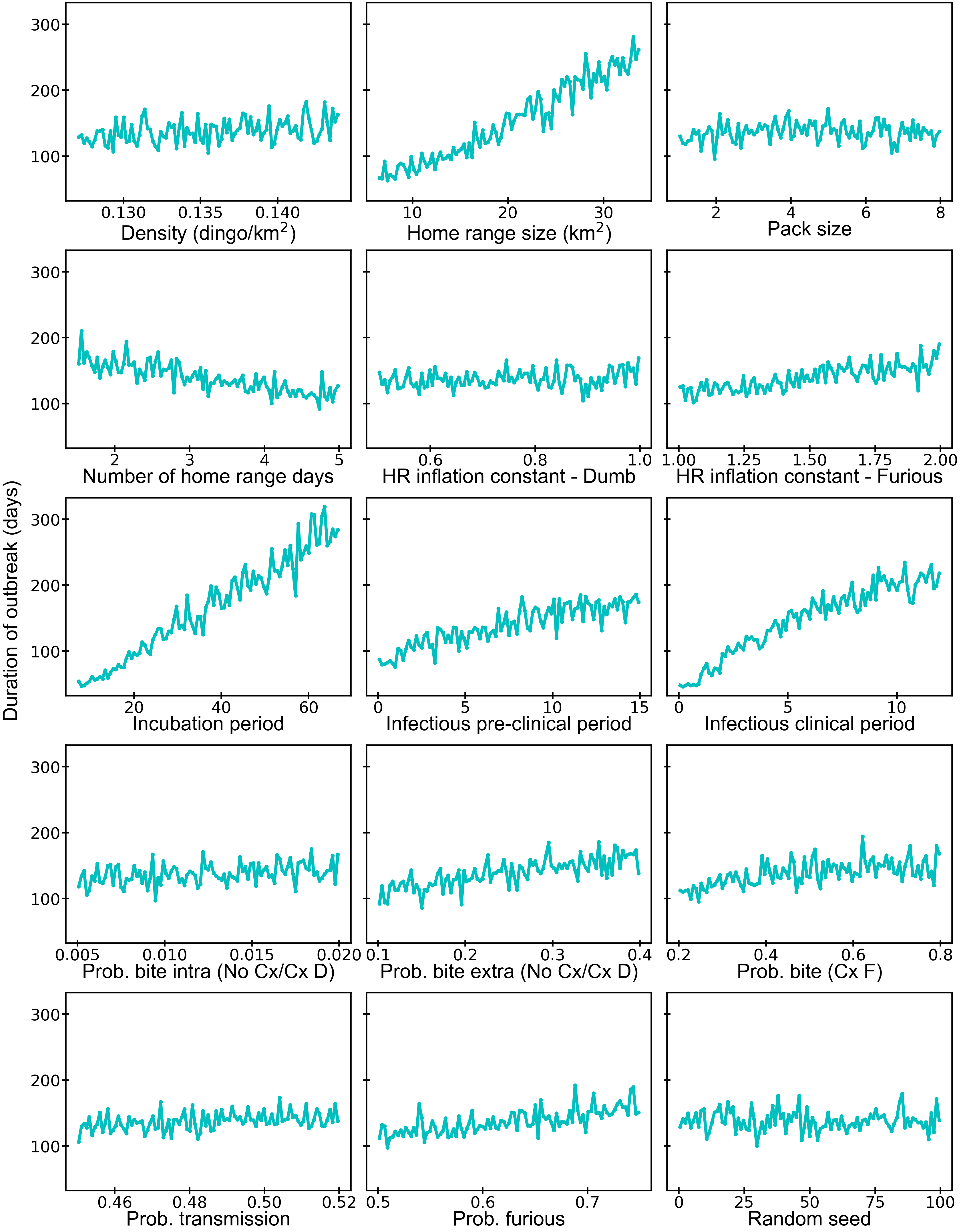

Supplement: S10 Fig — (See legend of S5 Fig). (TIF) [file pntd.0009124.s012.tif]

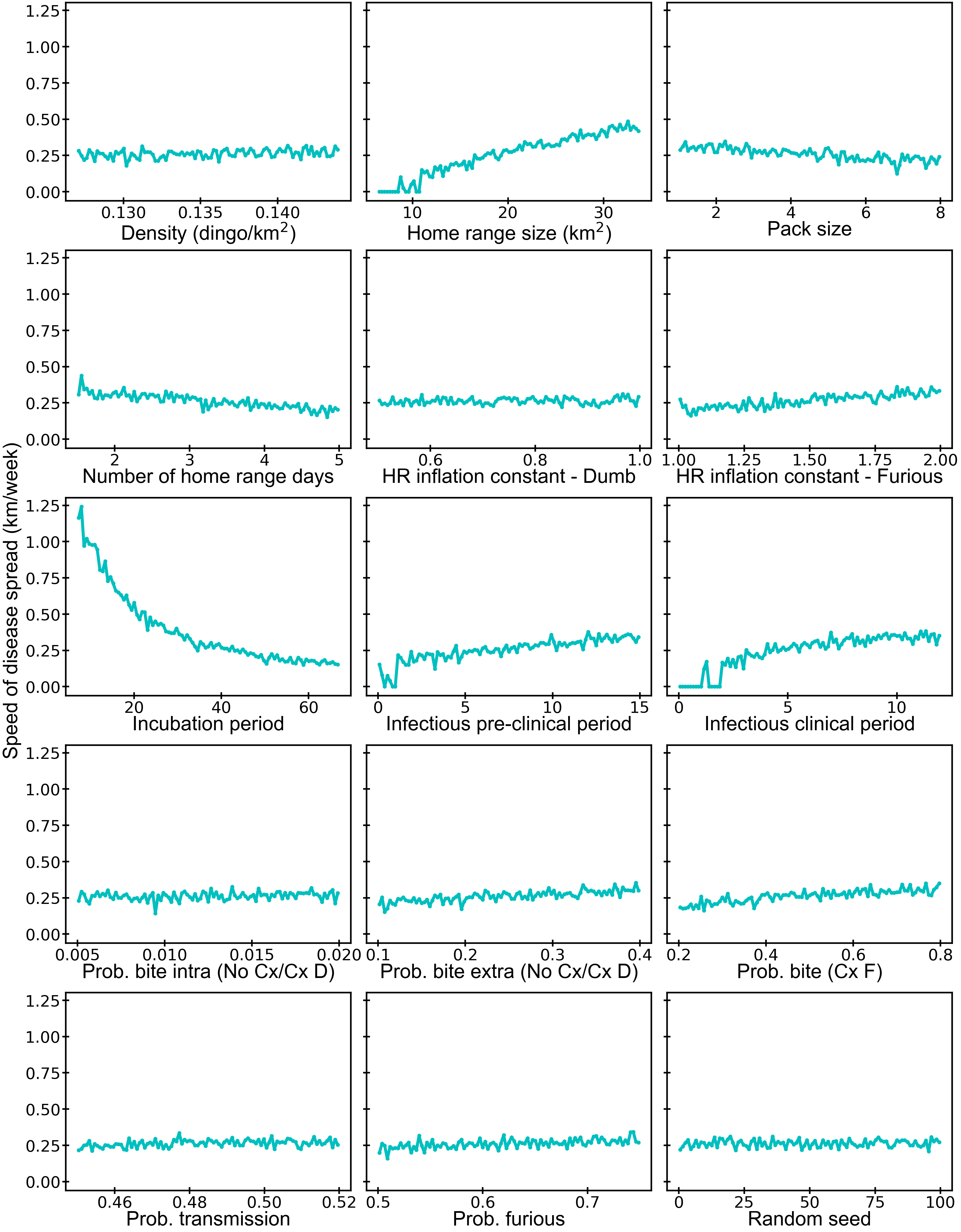

Supplement: S11 Fig — (See legend of S5 Fig). (TIF) [file pntd.0009124.s013.tif]

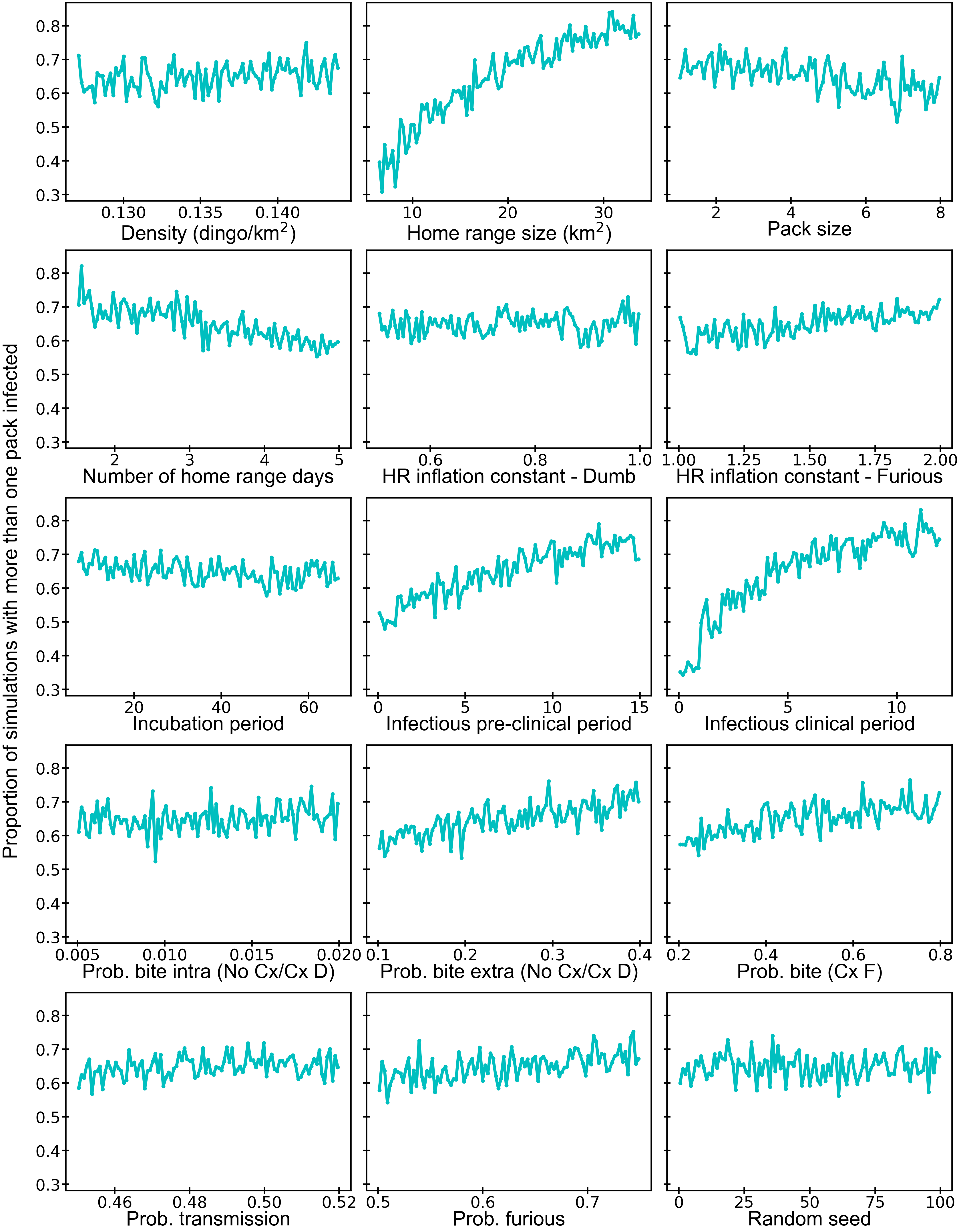

Supplement: S12 Fig — (See legend of S5 Fig). (TIF) [file pntd.0009124.s014.tif]

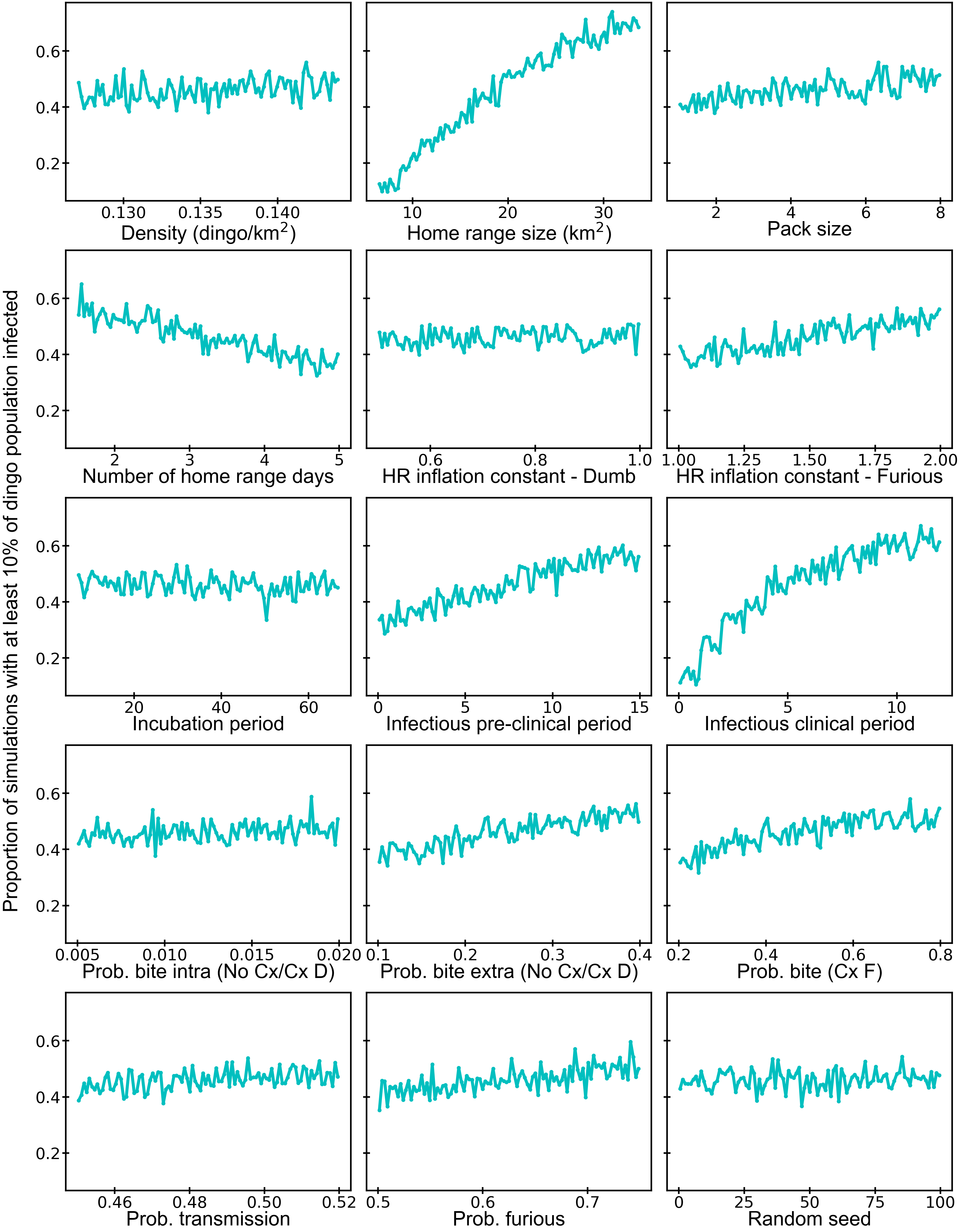

Supplement: S13 Fig — (See legend of S5 Fig). (TIF) [file pntd.0009124.s015.tif]
